# LSTMCNNsucc: A Bidirectional LSTM and CNN-Based Deep Learning Method for Predicting Lysine Succinylation Sites

**DOI:** 10.1155/2021/9923112

**Published:** 2021-05-28

**Authors:** Guohua Huang, Qingfeng Shen, Guiyang Zhang, Pan Wang, Zu-Guo Yu

**Affiliations:** ^1^School of Information Engineering, Shaoyang University, Shaoyang 42200, China; ^2^Key Laboratory of Intelligent Computing and Information Processing of Ministry of Education and Hunan Key Laboratory for Computation and Simulation in Science and Engineering, Xiangtan University, Xiangtan, Hunan 411105, China

## Abstract

Lysine succinylation is a typical protein post-translational modification and plays a crucial role of regulation in the cellular process. Identifying succinylation sites is fundamental to explore its functions. Although many computational methods were developed to deal with this challenge, few considered semantic relationship between residues. We combined long short-term memory (LSTM) and convolutional neural network (CNN) into a deep learning method for predicting succinylation site. The proposed method obtained a Matthews correlation coefficient of 0.2508 on the independent test, outperforming state of the art methods. We also performed the enrichment analysis of succinylation proteins. The results showed that functions of succinylation were conserved across species but differed to a certain extent with species. On basis of the proposed method, we developed a user-friendly web server for predicting succinylation sites.

## 1. Introduction

Protein post-translational modification (PTM) refers to the chemical interaction occurring prior to protein biosynthesis and after mRNAs are translated into polypeptide chains. PTM has different categories and is very prevalent in the cells. More than 450 categories of PTMs were discovered to date, such as phosphorylation, methylation, and acetylation [1–3]. PTM increases diversity of protein structures and functions, viewed as one of most regulating mechanisms in the cellular process. Lysine succinylation is a type of protein TPMs, in which a succinyl group (-CO-CH2-CH2-CO2H) is attached to lysine residue of proteins [4]. Succinylation is reversible, dynamic, and evolutionarily conserved, widely existing in the prokaryote and the eukaryotes cells [5, 6]. The succinylation of proteins induces shift in the charge and the structural alteration and thus would yield effects on functions of proteins [6]. Growing evidences also showed aberrant succinylations were involved in the pathogenesis of some diseases including cancers [7], metabolism disease [8, 9], and nervous system diseases [10]. Thus, identifying succinylation sites and understanding its mechanism are crucial to develop drugs for related diseases.

Identifying succinylation sites has two main routes: experimental and computational methods. The experimental methods were represented by mass spectrometry, which contributed to the validation of succinylation and collection of first-hand data. On the other hand, the experimental methods are labor-intensive and time-consuming without assist of the computational methods. The computational methods are based on data yielded by the experimental methods and build machine learning-based models to predict new succinylations. Therefore, identifying succinylation is a cyclic iterative process from experiment to computation and again from computation to experiment. We focused on the computational methods to predict succinylation. In the past decades, more than ten computational methods have been developed for identifying succinylation [11–29]. Most of these computational methods extracted features directly from protein sequences, which were subsequently used for training model. For example, Zhao et al. [11] used the auto-correlation functions, the group weight-based encoding, the normalized van der Waals volume, and the position weight amino acid composition. Kao et al. [25] exploited the amino acid composition and informative *k*-spaced amino acid pairs. Xu et al. [12] and Jia et al. [13, 19] employed pseudo amino acid composition. Dehzangi et al. [23] exploited the structure information. Hasan et al. [28] compared 12 types of feature as well as two learning methods: random forest and support vector machine for succinylation prediction. Different features have different performance with species. So does the learning methods. The best performance was no more than 0.83 AUC (area under receiver operating characteristic curve) for independent test. Like sentences of language, the protein sequences should have semantic. However, all the methods above failed to seize semantic relationship hidden among residues. Thapa et al. [29] presented a convolutional neural network- (CNN-) based deep learning method DeepSuccinylSite for predicting succinylation. Different from traditional methods, the DeepSuccinylSite exploited word embedding which translated word into vector, which was an extensively used method in the field of natural language process. The CNN is a widely used method to extract local features especially in the field of image processing. Inspired by the DeepSuccinylSite and loss of semantic relationship between residues, we fused long short-term memory (LSTM) and CNN into a deep learning method for succinylation prediction.

## 2. Data

All the succinylated proteins were downloaded from the PLMD (Protein Lysine Modifications Database) database which is dedicated to specifically collect protein lysine modification [30–32]. The PLMD has evolved to version 3.0, housing 284780 modification events in 53501 proteins for 20 types of lysine modification. We extracted 6377 proteins containing 18593 succinylation sites. To remove dependency of the proposed method on the homology, we used the software CD-Hit [33, 34] to cluster 6377 protein sequences. The sequence identify cut-off was set to 0.4, and we obtained 3560 protein sequences, of which any two kept sequence similarity less than 0.4. We randomly divided these 3560 proteins into the training and the testing samples at the ratio of training to testing 4 : 1, resulting in 712 testing and 2848 training sequences. For each protein sequence, we extracted all the peptides which centered the lysine residue with 15 amino acid residues in the downstream/upstream of it. For peptides less than 15 amino acid residues, we prefixed or suffixed “*X*” to supply it. The length of the amino acids is influential in prediction of succinylation sites. The short amino acid peptides would miss key information, while the long peptides would include noise or redundancy. Whether the short or the long peptides would cause low accuracy of prediction. Among methods to predict succinylation sites, iSuc-PseAAC [12] adopted the shorter peptides of 15 amino acid residues; SuccinSite2.0 [20] and GPSuc [22] adopted the longer 41 amino acid residues, while the most methods including SSEvol-Suc [23], Success [24], iSuc-PseOpt [13], pSuc-Lys [19], SucStruct [18], and PSSM-Suc [17] adopted peptides of 31 amino acid residues, which is of moderate length. Thus, we chose 31 amino acid residues as basic peptides. The peptides with succinylation sites were viewed positive samples and the others as negative ones. For the training set, the negative samples extremely outnumbered the positive ones. Unbalanced training set would cause preference to negative samples in the process of prediction. Therefore, we randomly sampled the same size of negative examples as the positive ones. Finally, the training set comprised 6512 positive and 6512 negative samples, while the testing set 1479 positive and 16457 negative samples. All the experimental data are freely available to scientific communities.

## 3. Method

**Figure 1:**
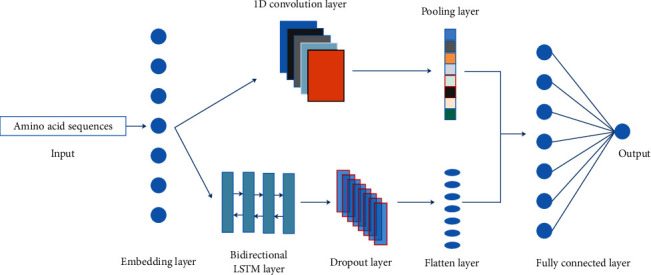
Flowchart of the proposed method.

As shown in Figure 1, the proposed deep learning network consisted mainly of embedding, 1D convolution, pooling, bidirectional LTSM, dropout, flatten, and fully connected layers. Peptides with 31 amino acid residues were entered to the embedding layer and were translated into vectors with shape of (31, 64). Then, two different network structures, respectively, took the embedding as input, and their outputs were concatenated as input to the fully connected layer. One structure was the convolution neural network, and another was the bidirectional LSTM neural network. The final output was a neuron representing probability of belonging to the positive sample. The parameters and the shape of output of each layers in the deep neural network are listed in Table 1. The total number of trainable parameters is 336,897.

### 3.1. Embedding Layer

Most machine learning-based methods for predicting protein post-translational modification generally required an encoding step which translated sequences into vector representation. For example, the frequently used encoding schemes included position specific scoring matrix [35], amino acid composition, composition of *k*-space amino acid pair [14], and pseudo amino acid composition [36]. For sequences of text, these methods might lose hidden semantic. The word2vec [37, 38] is different from the above methods, embedding word into vector. The word2vec is capable of extracting semantic of word. An interesting example is that King–Man + Woman = Queen. Similar to the word2vec [37, 38], the embedding layer translated words into vector representations. In this method, the character of amino acid corresponds to word.

### 3.2. 1D Convolution Layer

The convolution neural network (CNN) proposed by LeCun et al. [39, 40] is a feed forward network. Compared with the conventional neural network, the CNN has two notable properties: local connectivity and parameter sharing. The local connectivity lies that two neighboring layers are not fully connected but locally connected. That is to say, the neuron in a layer is not connected to all neurons in the neighboring layers. The CNN implemented the parameter sharing via the filter (also called convolution kernel). The filter slides on the image and convoluted with all sections in image. The filter is shared by the image. In the last ten years, many deep convolution neural networks such as AlexNet [41], VGG [42], GoogleNet [43], and ResNet [44] have been proposed and applied to computer vision. The CNN achieved significant advance in terms of classification error in comparison with the previous deep neural network. The convolution is of 1-dimension, 2-dimension, or more than 2 dimensions. Here, we used 1D convolution. Suppose a discrete sequence was *α* = [*a*_1_, *a*_2_, ⋯, *a*_*n*_], and the convolution kernel was *β* = [*b*_1_, *b*_2_, ⋯*b*_*m*_]. The 1D convolution product of *α* and *β* was expressed by

(1) $$\begin{array}{c} \alpha *\beta =\left[\overset{m}{\underset{i=1}{\sum }}a_{jd+i-1}b_{i}\right],j=1,2,\cdots ,k, \end{array}$$

where *d* was the stride of convolution and *k* was the length of the output sequence. Generally, *k* was the most integer less than or equal to (*n* − *m*)/*d* + 1.

### 3.3. Pooling Layer

The pooling operation firstly appeared in the AlexNet [41] and is increasingly becoming one of components of the deep CNN architecture. The pooling operation has such categories as max pooling, min pooling, and mean pooling. The role of pooling operation included removal of redundancy information and reduction of overfitting. Here, we used the max pooling operation. Given an *n*-channel input *A* = (*a*_*i*,*j*,*k*_), the max pooling operation was defined by

(2) $$\begin{array}{c} \underset{j}{\max}\left\{a_{i,j,k}\right\}. \end{array}$$

### 3.4. Bidirectional LSTM Layer

**Figure 2:**
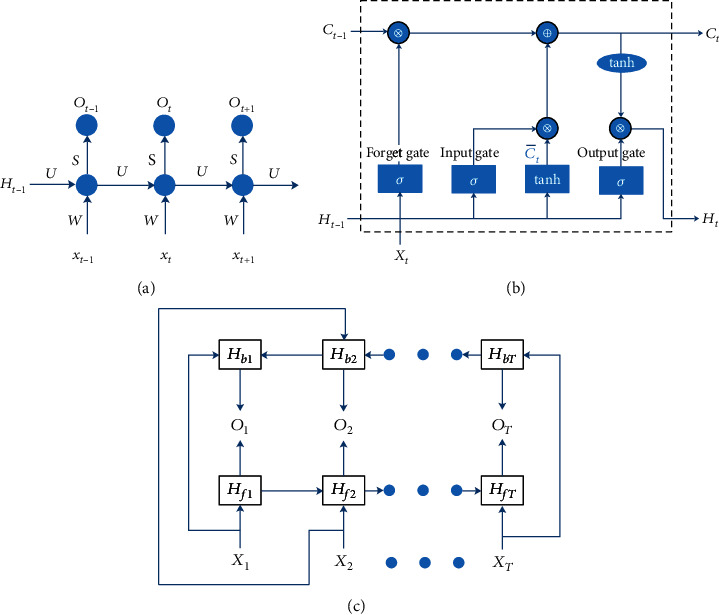
The structure of neural networks: (a) for RNN, (b) for LSTM, and (c) for directional LSTM.

Recurrent neural network (RNN) [45, 46] is a different framework of neural network from multiple layer perception. The RNN shares weights and is especially suitable to the field of sequence analysis such as language translation and semantic understanding. An unfolded RNN model was shown in Figure 2(a). The hidden state *H*_*t*_ at the time step *t* was not only dependent on the current input but also on the previous hidden state, which was computed by

(3) $$\begin{array}{c} H_{t}=f\left(X_{t}W+H_{t-1}U+\alpha \right), \end{array}$$

where *f* was an activation function and *α* was a bias. The output *O*_*t*_ at the time step *t* was computed by

(4) $$\begin{array}{c} O_{t}=g\left(H_{t}S+\beta \right), \end{array}$$

where *g* was also an activation function and *β* was a bias. For long sequences, there was a fatal question with the RNN, i.e., vanishing gradient. Among all the solutions to the vanishing gradient, the LSTM [47] is one of the better. The LSTM contains a candidate memory cell and three gates: forget gate, input gate, and output gate, as shown in Figure 2(b). The forget gate *F*_*t*_, the input gate *I*_*t*_, and the output gate *P*_*t*_ at the time step *t* were computed, respectively, by

(5) $$\begin{array}{c} F_{t}=\sigma \left(X_{t}W_{x,f}+H_{t-1}W_{h,f}+b_{f}\right),\\ I_{t}=\sigma \left(X_{t}W_{x,i}+H_{t-1}W_{h,i}+b_{i}\right),\\ P_{t}=\sigma \left(X_{t}W_{x,o}+H_{t-1}W_{h,o}+b_{o}\right), \end{array}$$

where *W*_*x*,*f*_ and *W*_*h*,*f*_ were weights of the LSTM from input to forget gate and from the hidden state to the forget gate, respectively. *W*_*x*,*i*_ and *W*_*h*,*i*_ were link weights from input to input gate and from the hidden state to the input gate, respectively. *W*_*x*,*o*_ and *W*_*h*,*o*_ were link weights from input to output gate and from the hidden state to the output gate, respectively. *b*_*f*_, *b*_*i*_, and *b*_*o*_ were the bias of the forget and the input and the output gate, respectively. *σ* was the activation function. The candidate memory cell was calculated by

(6) $$\begin{array}{c} {\bar{C}}_{t}=\tanh\left(X_{t}W_{x,c}+H_{t-1}W_{h,c}+b_{c}\right), \end{array}$$

where *W*_*x*,*c*_ and *W*_*h*,*c*_ were weights of the LSTM from input to the candidate memory and from the hidden state to the candidate memory, respectively, and *b*_*c*_ was the bias. The memory cell at the time step *t* was computed by

(7) $$\begin{array}{c} C_{t}=F_{t}⨂C_{t-1}+I_{t}⨂{\bar{C}}_{t}, \end{array}$$

where ⨂ was defined as element-wise multiplication. The hidden state was updated by

(8) $$\begin{array}{c} H_{t}=I_{t}⨂\tanh\left(C_{t}\right). \end{array}$$

The previous RNN was forward. The output at the time step *t* was only dependent on the preceding inputs and the hidden state. In fact, the output might be relevant to the latter input and the hidden state. Schuster et al. [48] proposed a bidirectional RNN to model this relationship, showed in Figure 2(c). The forward hidden state at the time step *t* was computed by

(9) $$\begin{array}{c} H_{t}^{f}=\sigma \left(X_{t}W_{x,h}^{f}+H_{t-1}^{f}W_{h,h}^{f}+b_{h}^{f}\right), \end{array}$$

while the backward hidden state was computed by

(10) $$\begin{array}{c} H_{t}^{b}=\sigma \left(X_{t}W_{x,h}^{b}+H_{t+1}^{b}W_{h,h}^{b}+b_{h}^{b}\right). \end{array}$$

The output at the time step *t* was computed by

(11) $$\begin{array}{c} O_{t}=\left[H_{t}^{f},H_{t}^{b}\right]W_{h,o}+b_{o}. \end{array}$$

### 3.5. Dropout Layer

The deep neural network is prone to lead to overfitting when the number of training samples was too less. To deal with this issue, Hinton et al. [49] proposed the dropout concept. Due to its effect and efficiency, the dropout is increasingly becoming the frequently used trick in the deep learning area [41, 50–53]. The neurons were dropped out at a certain rate of dropout, and parameters of only preserved neurons were updated in the training stage, while all the neurons were used in the predicting stage.

### 3.6. Flatten Layer and Fully Connected Layer

The role of flatten layer was only to convert the data into one-dimension and then facilitated connection of the fully connected layer. No parameters were trainable in the flatten layer. The fully connected layer was similar to hidden layer in the MLP, each neuron connected to the neurons in the preceding layer.

## 4. Metrics

We adopted to evaluate the predicted result these frequently used metrics in the binary classification questions such as sensitivity (SN), specificity (SP), accuracy (ACC), and Matthews correlation coefficient (MCC), which were defined by

(12) $$\begin{array}{c} \text{SN}=\frac{\text{TP}}{\text{TP}+\text{FN}},\\ \text{SP}=\frac{\text{TN}}{\text{FP}+\text{TN}},\\ \text{ACC}=\frac{\text{TP}+\text{TN}}{\text{TP}+\text{FN}+\text{FP}+\text{TN}},\\ \text{MCC}=\frac{\text{TP}\times \text{TN}-\text{FP}\times \text{FN}}{\sqrt{\left(\text{TP}+\text{FN}\right)\left(\text{TP}+\text{FP}\right)\left(\text{TN}+\text{FN}\right)\left(\text{TN}+\text{FP}\right)}}, \end{array}$$

where TP and TN were defined as numbers of the true positive and the true negative samples, respectively, FP and FN, respectively, as numbers of the false positive and the false negative samples in the prediction. SN reflected the accuracy of the correctly predicted positive samples, SP accuracy of the correctly predicted negative samples, and ACC the average accuracy of the correctly predicted samples. SN, SP, and ACC ranged from 0 to 1, larger meaning better performance. MCC was Matthews correlation coefficient, representing correlation between the true class and the predicted class. MCC ranged from -1 to 1. 1 meant perfect prediction, 0 random prediction, and -1 meant that the prediction was completely opposite to the true.

## 5. Results

Table 2 showed the predicting performance of the trained model on the 712 testing sequences. Although more than ten approaches or tools for predicting succinylation have been proposed in the past ten years, either they did not provide online predicting server or the web server could not work. We compared the proposed method to three methods whose web predicting server still can work [28]: SuccinSite [15], iSuc-PseAAC [12], and DeepSuccinylSite [29]. 712 testing sequences were used to examine three approaches. Among 712 testing sequences, at least 225 sequences repeated in the training set of the SuccinSite, and at least 223 repeated in the training set of DeepSuccinylSite. These minus 225 sequences were used to examine the SuccinSite and these minus 223 sequences to test the DeepSuccinylSite. iSuc-PseAAC [12] obtained best SP and best ACC but worst SN and worst MCC. The SuccinSite [15] reached better SP and better ACC but worse MCC and worse SN. The iSuc-PseAAC [12] and the SuccinSite [15] were in favor of predicting the negative samples. The DeepSuccinylSite [29] was better than the LSTMCNNsucc in terms of SN, worse than the LSTMCNNsucc in terms of sp. The overall performance of the LSTMCNNsucc was slightly better than that of the DeepSuccinylSite.

### 5.1. Functional Analysis

**Figure 3:**
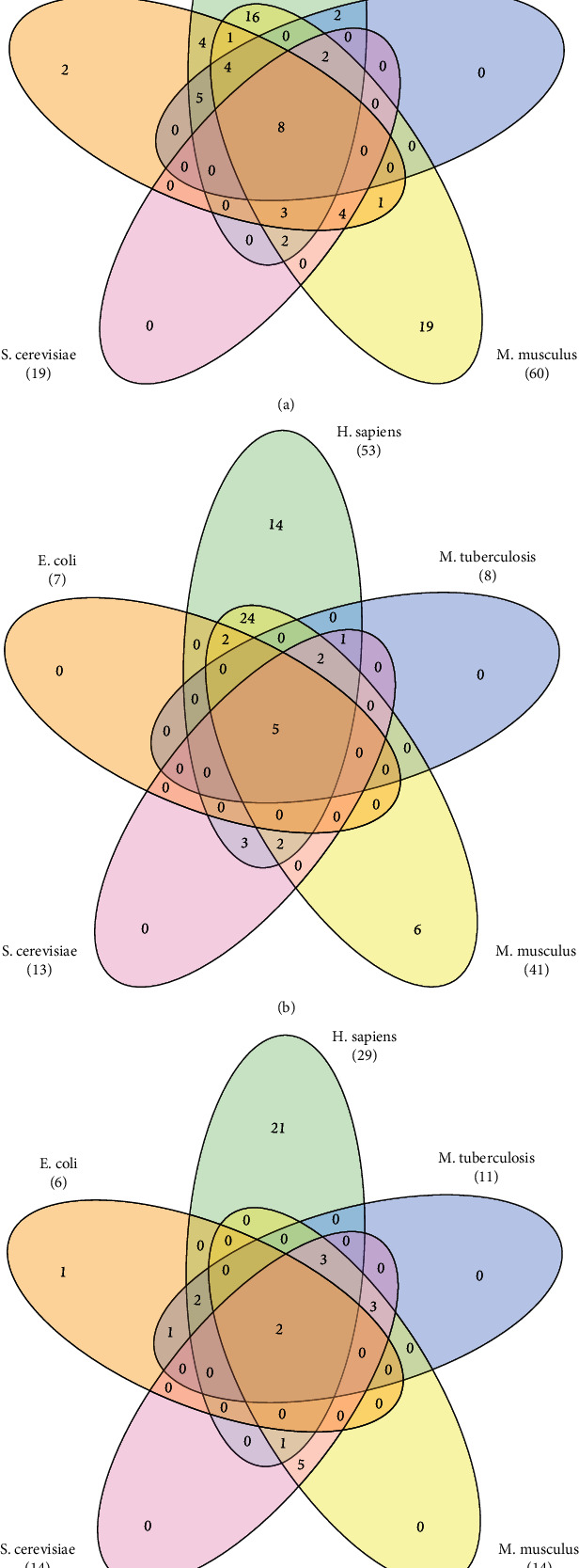
The numbers of shared terms (a) for biological process, (b) cellular component, and (c) molecular function.

We used the statistical over-representation test of gene list analysis in the PANTHER classification system [54, 55] to perform function enrichment analysis of the succinylated proteins. The significant biological process, the molecular function, and the cellular component terms (*p* value≤0.01) were listed in the supplementary materials 1 and 2. For five species, Escherichia coli (E. coli), Homo sapiens (H. sapiens), Mus musculus (M. musculus), Mycobacterium tuberculosis (M. tuberculosis), and Saccharomyces cerevisiae (S. cerevisiae), they shared some common functions, but they had also own specific functions. The numbers of shared terms among five species are shown in Figure 3. H. sapiens and M. musculus shared 36 significant biological process terms and 35 cellular component terms, much more than the numbers of shared terms between any other two species (Figures 3(a) and 3(b)). Five species shared eight biological process GO terms: “biosynthetic process (GO:0009058)”, “carboxylic acid metabolic process (GO:0019752)”, “organic acid metabolic process (GO:0006082)”, “organic substance biosynthetic process (GO:1901576)”, “organonitrogen compound biosynthetic process (GO:1901566)”, “organonitrogen compound metabolic process (GO:1901564)”, “oxoacid metabolic process (GO:0043436)”, and “small molecule metabolic process (GO:0044281)”; 5 cellular component GO terms: “cytoplasm (GO:0005737)”, “cytoplasmic part (GO:0044444)”, “cytosol (GO:0005829)”, “intracellular (GO:0005622)”, and “intracellular part (GO:0044424)”; and two molecular function GO terms: “catalytic activity (GO:0003824)”, and “molecular_function (GO:0003674)”. H. sapiens had much more own specific functions than other species, with 75 specific biological process GO terms, 14 GO cellular component terms, and 21 molecular function GO terms. No specific functions existed in both M. tuberculosis and S. cerevisiae whether for biological process, cellular component, or molecular functions.

We also performed enrichment analysis of Kyoto Encyclopedia of Genes and Genomes (KEGG) pathway by functional annotation in the DAVID tool [56, 57] to investigate in which pathway the succinylated proteins were involved. The statistically significant KEGG terms (Benjamini ≤ 0.01) are listed in Table 3. Different species were involved in some identical pathways. For example, both metabolic pathways and biosynthesis of antibiotics were enriched in the succinylated proteins for five species, implying the universal role of succinylation. On the other hand, different pathways were involved in different species. H. sapiens and M. musculus shared more pathway and had more pathways than other three species, implying species-specific role of the succinylation.

### 5.2. LSTMCNNsucc Web Server

We built a web server of the proposed LSTMCNNsucc at http://8.129.111.5/. Users either directly input protein sequences in a fasta format or upload a file of fasta format to perform prediction. When both protein sequences and files were submitted, the file was given to priority of prediction.

## 6. Conclusion

We presented a bidirectional LSTM and CNN-based deep learning method for predicting succinylation sites. The method absorbed semantic relationship hidden in the succinylation sequences, outperforming state-of-the-art method. The functions of succinylation proteins were conserved to a certain extent across species but also were species-specific. We also implemented the proposed method into a user-friendly web server which is available at http://8.129.111.5/.

## Figures and Tables

**Table 1:** Number of parameters and shape of output in the LSTMCNNsucc.

Layers	Parameters	Output
Embedding	1472	(None, 31, 64)
Bidirectional LSTM	197632	(None, 31, 256)
Dropout	0	(None, 31, 256)
Flatten	0	(None, 7936)
1D convolution	10272	(None, 27, 32)
Pooling	0	(None, 32)
Dense (16)	127504	(None, 16)
Dense (1)	17	(None, 1)

**Table 2:** Comparison with state of the art methods.

Method	SN	SP	ACC	MCC
LSTMCNNsucc	0.5916	0.7957	0.7789	0.2508
SuccinSite [15]	0.3977	0.8635	0.8272	0.1925
iSuc-PseAAC [12]	0.1258	0.8929	0.8296	0.0165
DeepSuccinylSite [29]	0.7438	0.6879	0.6923	0.2438

**Table 3:** Significant KEGG pathway terms.

Species	KEGG terms	Benjamini
E. coli	Metabolic pathways	3.30*E*-08
	Biosynthesis of amino acids	1.00*E*-06
	Biosynthesis of secondary metabolites	2.40*E*-04
	Biosynthesis of antibiotics	7.40*E*-04
	Lysine biosynthesis	3.30*E*-03

H. sapiens	Biosynthesis of antibiotics	3.70*E*-10
	Metabolic pathways	2.80*E*-09
	Ribosome	3.40*E*-08
	Valine, leucine, and isoleucine degradation	1.30*E*-06
	Carbon metabolism	6.20*E*-06
	Oxidative phosphorylation	1.10*E*-05
	Parkinson's disease	2.60*E*-05
	Citrate cycle (TCA cycle)	1.00*E*-04
	Huntington's disease	4.10*E*-04
	Alzheimer's disease	7.80*E*-04
	Aminoacyl-tRNA biosynthesis	1.00*E*-03
	Butanoate metabolism	3.40*E*-03
	Proteasome	8.20*E*-03

M. musculus	Metabolic pathways	6.20*E*-26
	Parkinson's disease	8.50*E*-11
	Oxidative phosphorylation	3.40*E*-10
	Nonalcoholic fatty liver disease (NAFLD)	1.00*E*-09
	Huntington's disease	2.80*E*-09
	Alzheimer's disease	1.40*E*-08
	Ribosome	3.30*E*-07
	Peroxisome	1.80*E*-06
	Glycine, serine, and threonine metabolism	1.50*E*-05
	Pyruvate metabolism	9.00*E*-05
	Propanoate metabolism	2.40*E*-04
	Valine, leucine, and isoleucine degradation	1.90*E*-03
	Glyoxylate and dicarboxylate metabolism	3.10*E*-03
	Biosynthesis of antibiotics	5.60*E*-03

M. tuberculosis	Metabolic pathways	1.00*E*-04
	Microbial metabolism in diverse environments	2.50*E*-04
	Biosynthesis of antibiotics	4.40*E*-04
	Biosynthesis of secondary metabolites	1.00*E*-02
	Propanoate metabolism	1.00*E*-02

S. cerevisiae	Metabolic pathways	5.20*E*-05
	Biosynthesis of amino acids	3.30*E*-04
	2-Oxocarboxylic acid metabolism	7.90*E*-04
	Biosynthesis of antibiotics	3.50*E*-03
	Oxidative phosphorylation	3.50*E*-03

## Data Availability

The experimental succinylation and nonsuccinylation sites used to support the findings of this study have been deposited in the website http://8.129.111.5/ and are freely available to all scientific communities.

## Supplementary Materials

Supplementary 1 KEGG enrichmeent analysis of KEGG pathway for 5 species.

Supplementary 2 GO enrichment analysis of GO terms for 5 species.
