# Supplementary material for: LSTMCNNsucc: A Bidirectional LSTM and CNN-Based Deep Learning Method for Predicting Lysine Succinylation Sites
Source: Biomed Res Int. 2021 May 28;2021:9923112. doi: 10.1155/2021/9923112 (PMC8188601; doi:10.1155/2021/9923112)
Supplement: Supplementary 1 — KEGG enrichmeent analysis of KEGG pathway for 5 species. [file 9923112.f1.pdf]

Category	EC<br>Term	Count	%	P-Value	Benjamini
KEGGPathway	Metabolic pathways	234	29.4	3.30E-10	3.30E-08
KEGGPathway	Biosynthesis of amino acids	59	7.4	2.00E-08	1.00E-06
KEGGPathway	Biosynthesis of secondary metabolites	108	13.6	7.10E-06	2.40E-04
KEGGPathway	Biosynthesis of antibiotics	76	9.5	3.00E-05	7.40E-04
KEGGPathway	Lysine biosynthesis	12	1.5	1.70E-04	3.30E-03
KEGGPathway	2-Oxocarboxylic acid metabolism	15	1.9	2.20E-03	3.70E-02

homo-sapiens

Category	Term	Count	%	P-Value	Benjamini
KEGG_PATHWAY	Biosynthesis of antibiotics	32	8	2.00E-12	3.70E-10
KEGG_PATHWAY	Metabolic pathways	83	20.6	2.90E-11	2.80E-09
KEGG_PATHWAY	Ribosome	23	5.7	5.50E-10	3.40E-08
KEGG_PATHWAY	Valine, leucine and isoleucine degradation	13	3.2	2.70E-08	1.30E-06
KEGG_PATHWAY	Carbon metabolism	18	4.5	1.60E-07	6.20E-06
KEGG_PATHWAY	Oxidative phosphorylation	19	4.7	3.60E-07	1.10E-05
KEGG_PATHWAY	Parkinson's disease	19	4.7	9.80E-07	2.60E-05
KEGG_PATHWAY	Citrate cycle (TCA cycle)	9	2.2	4.30E-06	1.00E-04
KEGG_PATHWAY	Huntington's disease	20	5	2.00E-05	4.10E-04
KEGG_PATHWAY	Alzheimer's disease	18	4.5	4.10E-05	7.80E-04
KEGG_PATHWAY	Aminoacyl-tRNA biosynthesis	11	2.7	5.80E-05	1.00E-03
KEGG_PATHWAY	Butanoate metabolism	7	1.7	2.10E-04	3.40E-03
KEGG_PATHWAY	Proteasome	8	2	5.70E-04	8.20E-03
KEGG_PATHWAY	Fatty acid degradation	7	1.7	2.50E-03	3.30E-02
KEGG_PATHWAY	Biosynthesis of amino acids	9	2.2	2.60E-03	3.30E-02
KEGG_PATHWAY	Synthesis and degradation of ketone bodies	4	1	3.70E-03	4.40E-02
KEGG_PATHWAY	DNA replication	6	1.5	6.50E-03	7.20E-02

mus-musculus

Category	Term	Count	%	P-Value	Benjamini
KEGG_PATHWAY	Metabolic pathways	138	25.4	2.80E-28	6.20E-26
KEGG_PATHWAY	Parkinson's disease	31	5.7	7.60E-13	8.50E-11

KEGG_PATHWAY	Oxidative phosphorylation	29	5.3	4.60E-12	3.40E-10
KEGG_PATHWAY	Non-alcoholic fatty liver disease (NAFLD)	30	5.5	1.80E-11	1.00E-09
KEGG_PATHWAY	Huntington's disease	33	6.1	6.20E-11	2.80E-09
KEGG_PATHWAY	Alzheimer's disease	30	5.5	3.70E-10	1.40E-08
KEGG_PATHWAY	Ribosome	25	4.6	1.00E-08	3.30E-07
KEGG_PATHWAY	Peroxisome	18	3.3	6.30E-08	1.80E-06
KEGG_PATHWAY	Glycine, serine and threonine metabolism	12	2.2	6.20E-07	1.50E-05
KEGG_PATHWAY	Pyruvate metabolism	11	2	4.10E-06	9.00E-05
KEGG_PATHWAY	Propanoate metabolism	9	1.7	1.20E-05	2.40E-04
KEGG_PATHWAY	Valine, leucine and isoleucine degradation	11	2	1.00E-04	1.90E-03
KEGG_PATHWAY	Glyoxylate and dicarboxylate metabolism	8	1.5	1.80E-04	3.10E-03
KEGG_PATHWAY	Biosynthesis of antibiotics	22	4.1	3.50E-04	5.60E-03
KEGG_PATHWAY	Fatty acid metabolism	9	1.7	1.40E-03	2.10E-02
KEGG_PATHWAY	Arginine and proline metabolism	8	1.5	4.70E-03	6.50E-02
KEGG_PATHWAY	Aminoacyl-tRNA biosynthesis	9	1.7	7.20E-03	9.40E-02
KEGG_PATHWAY	One carbon pool by folate	5	0.9	7.90E-03	9.70E-02

Mycobacterium tuberculosis

Category	Term	Count	%	P-Value	Benjamini
KEGG_PATHWAY	Metabolic pathways	170	30.4	1.00E-06	1.00E-04
KEGG_PATHWAY	Microbial metabolism in diverse environments	74	13.2	5.00E-06	2.50E-04
KEGG_PATHWAY	Biosynthesis of antibiotics	79	14.1	1.30E-05	4.40E-04
KEGG_PATHWAY	Biosynthesis of secondary metabolites	93	16.6	5.00E-04	1.00E-02
KEGG_PATHWAY	Propanoate metabolism	24	4.3	5.00E-04	1.00E-02
KEGG_PATHWAY	Carbon metabolism	40	7.2	1.50E-03	2.40E-02
KEGG_PATHWAY	Valine, leucine and isoleucine degradation	25	4.5	1.60E-03	2.40E-02

KEGG_PATHWAY	Glyoxylate and dicarboxylate metabolism	19	3.4	2.40E-03	3.00E-02
KEGG_PATHWAY	Fatty acid metabolism	22	3.9	3.60E-03	4.00E-02
KEGG_PATHWAY	Biosynthesis of amino acids	40	7.2	4.00E-03	4.00E-02
KEGG_PATHWAY	Ribosome	24	4.3	6.50E-03	6.00E-02
KEGG_PATHWAY	Fatty acid degradation	21	3.8	8.60E-03	7.30E-02

Saccharomyces cerevisiae

Category	Term	Count	%	P-Value	Benjamini
KEGG_PATHWAY	Metabolic pathways	82	27.5	6.60E-07	5.20E-05
KEGG_PATHWAY	Biosynthesis of amino acids	25	8.4	8.10E-06	3.30E-04
KEGG_PATHWAY	2-Oxocarboxylic acid metabolism	12	4	2.90E-05	7.90E-04
KEGG_PATHWAY	Biosynthesis of antibiotics	32	10.7	2.10E-04	3.50E-03
KEGG_PATHWAY	Oxidative phosphorylation	16	5.4	2.20E-04	3.50E-03
KEGG_PATHWAY	Biosynthesis of secondary metabolites	38	12.8	8.60E-04	1.10E-02
KEGG_PATHWAY	Amino sugar and nucleotide sugar metabolism	9	3	2.00E-03	2.30E-02
KEGG_PATHWAY	Proteasome	9	3	3.70E-03	3.70E-02
KEGG_PATHWAY	Valine, leucine and isoleucine biosynthesis	5	1.7	9.80E-03	8.70E-02
