# Supplementary material for: LSTMCNNsucc: A Bidirectional LSTM and CNN-Based Deep Learning Method for Predicting Lysine Succinylation Sites
Source: Biomed Res Int. 2021 May 28;2021:9923112. doi: 10.1155/2021/9923112 (PMC8188601; doi:10.1155/2021/9923112)
Supplement: Supplementary 2 — GO enrichment analysis of GO terms for 5 species. [file 9923112.f2.pdf]

Analysis Type:	PANTHER Overrepresentation Test (Released 20200728)
Annotation Version and Release Date:	PANTHER version 15.0 Released 2020-02-14
Analyzed List:	protein.txt (Escherichia coli)
Reference List:	Escherichia coli (all genes in database)
Test Type:	FISHER
Correction:	FDR

PANTHER GO-Slim Biological Process	Escherichia	protein.txt	protein.txt	protein.txt	protein.txt	protein.txt	protein.txt (FDR)
cellular metabolic process (GO:0044237)	727	208	121.86	+	1.71	4.82E-13	1.12E-10
metabolic process (GO:0008152)	797	222	133.59	+	1.66	4.55E-13	1.59E-10
organonitrogen compound metabolic process (GO:1901564)	326	118	54.64	+	2.16	1.64E-12	2.86E-10
organonitrogen compound biosynthetic process (GO:1901566)	215	92	36.04	+	2.55	4.16E-13	2.90E-10
nitrogen compound metabolic process (GO:0006807)	573	173	96.04	+	1.8	2.27E-12	3.16E-10

organic substance metabolic process (GO:0071704)	719	202	120.52	+	1.68	5.16E-12	6.00E-10
cellular biosynthetic process (GO:0044249)	412	133	69.06	+	1.93	3.96E-11	3.94E-09
biosynthetic process (GO:0009058)	420	134	70.4	+	1.9	5.75E-11	5.01E-09
organic substance biosynthetic process (GO:1901576)	409	130	68.55	+	1.9	1.88E-10	1.45E-08
cellular process (GO:0009987)	902	229	151.19	+	1.51	6.48E-10	4.52E-08
cellular nitrogen compound metabolic process (GO:0034641)	431	132	72.24	+	1.83	9.80E-10	6.21E-08
carboxylic acid biosynthetic process (GO:0046394)	86	46	14.41	+	3.19	2.79E-09	1.50E-07
small molecule metabolic process (GO:0044281)	329	107	55.15	+	1.94	3.20E-09	1.59E-07
organic acid biosynthetic process (GO:0016053)	86	46	14.41	+	3.19	2.79E-09	1.62E-07

small molecule biosynthetic process (GO:0044283)	131	58	21.96	+	2.64	4.28E-09	1.86E-07
primary metabolic process (GO:0044238)	606	166	101.58	+	1.63	4.15E-09	1.93E-07
oxoacid metabolic process (GO:0043436)	213	77	35.7	+	2.16	2.04E-08	8.35E-07
carboxylic acid metabolic process (GO:0019752)	209	76	35.03	+	2.17	2.53E-08	9.29E-07
organic acid metabolic process (GO:0006082)	215	77	36.04	+	2.14	2.49E-08	9.64E-07
organic cyclic compound metabolic process (GO:1901360)	400	114	67.05	+	1.7	4.02E-07	1.40E-05
biological_process (GO:0008150)	1188	267	199.13	+	1.34	5.58E-07	1.77E-05
Unclassified (UNCLASSIFIED)	3203	469	536.87	-	0.87	5.58E-07	1.85E-05
cellular amino acid biosynthetic process (GO:0008652)	65	33	10.9	+	3.03	1.14E-06	3.46E-05

heterocycle metabolic process (GO:0046483)	382	108	64.03	+	1.69	1.30E-06	3.62E-05
cellular nitrogen compound biosynthetic process (GO:0044271)	280	86	46.93	+	1.83	1.28E-06	3.73E-05
cellular amino acid metabolic process (GO:0006520)	115	46	19.28	+	2.39	1.83E-06	4.92E-05
cellular amide metabolic process (GO:0043603)	91	39	15.25	+	2.56	3.04E-06	7.86E-05
amide biosynthetic process (GO:0043604)	71	33	11.9	+	2.77	5.72E-06	1.42E-04
cellular aromatic compound metabolic process (GO:0006725)	388	106	65.03	+	1.63	7.39E-06	1.78E-04
alpha-amino acid metabolic process (GO:1901605)	81	34	13.58	+	2.5	1.98E-05	4.59E-04
organic cyclic compound biosynthetic process (GO:1901362)	234	69	39.22	+	1.76	4.56E-05	1.03E-03

heterocycle biosynthetic process (GO:0018130)	223	64	37.38	+	1.71	1.80E-04	3.91E-03
macromolecule metabolic process (GO:0043170)	374	94	62.69	+	1.5	3.99E-04	8.42E-03
gene expression (GO:0010467)	227	62	38.05	+	1.63	7.20E-04	1.48E-02
cofactor metabolic process (GO:0051186)	82	29	13.74	+	2.11	8.92E-04	1.78E-02
cofactor biosynthetic process (GO:0051188)	57	23	9.55	+	2.41	9.93E-04	1.92E-02
aromatic compound biosynthetic process (GO:0019438)	222	60	37.21	+	1.61	1.16E-03	2.19E-02
nucleobase-containing compound metabolic process (GO:0006139)	335	83	56.15	+	1.48	1.31E-03	2.41E-02
cell projection organization (GO:0030030)	43	0	7.21	-	< 0.01	1.83E-03	3.27E-02

monocarboxylic acid biosynthetic process (GO:0072330)	16	10	2.68	+	3.73	2.10E-03	3.66E-02
peptide biosynthetic process (GO:0043043)	55	21	9.22	+	2.28	2.45E-03	4.16E-02
RNA modification (GO:0009451)	44	18	7.38	+	2.44	2.76E-03	4.47E-02
monocarboxylic acid metabolic process (GO:0032787)	77	26	12.91	+	2.01	2.73E-03	4.53E-02
phosphorus metabolic process (GO:0006793)	107	33	17.93	+	1.84	3.09E-03	4.89E-02
translation (GO:0006412)	52	20	8.72	+	2.29	3.26E-03	4.93E-02
translational elongation (GO:0006414)	52	20	8.72	+	2.29	3.26E-03	5.04E-02

Analysis Type:	PANTHER Overrepresentation Test (Released 20200728)
Annotation Version and Release Date:	PANTHER version 15.0 Released 2020-02-14
Analyzed List:	protein.txt (Escherichia coli)
Reference List:	Escherichia coli (all genes in database)
Test Type:	FISHER
Correction:	FDR

PANTHER GO-Slim Cellular Component	Escherichia	protein.txt	protein.txt	protein.txt	protein.txt	protein.txt	protein.txt (FDR)
ribosomal subunit (GO:0044391)	27	12	4.53	+	2.65	8.94E-03	3.81E-02
intracellular organelle (GO:0043229)	46	20	7.71	+	2.59	1.05E-03	8.65E-03
ribosome (GO:0005840)	30	13	5.03	+	2.59	6.90E-03	3.17E-02
ribonucleoprotein complex (GO:1990904)	31	13	5.2	+	2.5	8.06E-03	3.57E-02
intracellular non-membrane-bounded organelle (GO:0043232)	36	15	6.03	+	2.49	4.44E-03	2.69E-02
organelle (GO:0043226)	53	22	8.88	+	2.48	6.89E-04	7.20E-03

outer membrane-bounded periplasmic space (GO:0030288)	44	18	7.38	+	2.44	2.76E-03	1.76E-02
periplasmic space (GO:0042597)	47	19	7.88	+	2.41	2.13E-03	1.44E-02
non-membrane-bounded organelle (GO:0043228)	43	17	7.21	+	2.36	4.52E-03	2.36E-02
organelle part (GO:0044422)	40	15	6.7	+	2.24	1.07E-02	4.41E-02
cytoplasmic part (GO:0044444)	349	120	58.5	+	2.05	1.68E-11	4.83E-10
cytoplasm (GO:0005737)	468	156	78.44	+	1.99	4.85E-14	1.86E-12
cytosol (GO:0005829)	319	106	53.47	+	1.98	1.34E-09	3.08E-08
intracellular part (GO:0044424)	490	161	82.13	+	1.96	3.74E-14	4.30E-12
intracellular (GO:0005622)	494	162	82.8	+	1.96	4.28E-14	2.46E-12
protein-containing complex (GO:0032991)	117	34	19.61	+	1.73	6.32E-03	3.03E-02
cell part (GO:0044464)	998	207	167.28	+	1.24	1.64E-03	1.26E-02
cell (GO:0005623)	998	207	167.28	+	1.24	1.64E-03	1.18E-02

cellular_component (GO:0005575)	1025	208	171.81	+	1.21	4.45E-03	2.44E-02
Unclassified (UNCLASSIFIED)	3366	528	564.19	-	0.94	4.45E-03	2.56E-02
cell periphery (GO:0071944)	467	43	78.28	-	0.55	2.56E-05	4.21E-04
plasma membrane (GO:0005886)	467	43	78.28	-	0.55	2.56E-05	3.68E-04
membrane (GO:0016020)	490	45	82.13	-	0.55	1.55E-05	2.98E-04
membrane part (GO:0044425)	219	20	36.71	-	0.54	5.91E-03	2.96E-02
intrinsic component of membrane (GO:0031224)	193	14	32.35	-	0.43	7.80E-04	6.90E-03
integral component of membrane (GO:0016021)	177	11	29.67	-	0.37	2.80E-04	3.58E-03
intrinsic component of plasma membrane (GO:0031226)	130	7	21.79	-	0.32	7.62E-04	7.30E-03
integral component of plasma membrane (GO:0005887)	125	6	20.95	-	0.29	5.44E-04	6.25E-03

Analysis Type:	PANTHER Overrepresentation Test (Released 20200728)
Annotation Version and Release Date:	PANTHER version 15.0 Released 2020-02-14
Analyzed List:	protein.txt (Escherichia coli)
Reference List:	Escherichia coli (all genes in database)
Test Type:	FISHER
Correction:	FDR

PANTHER GO-Slim Molecular Function	Escherichia	protein.txt	protein.txt	protein.txt	protein.txt	protein.txt	protein.txt (FDR)
nucleoside phosphate binding (GO:1901265)	47	19	7.88	+	2.41	2.13E-03	5.02E-02
nucleotide binding (GO:0000166)	47	19	7.88	+	2.41	2.13E-03	4.63E-02
kinase activity (GO:0016301)	69	26	11.57	+	2.25	9.15E-04	2.59E-02
ion binding (GO:0043167)	129	40	21.62	+	1.85	1.08E-03	2.78E-02
binding (GO:0005488)	358	107	60.01	+	1.78	1.55E-07	1.09E-05
nucleic acid binding (GO:0003676)	139	41	23.3	+	1.76	2.24E-03	4.53E-02
transferase activity (GO:0016740)	286	84	47.94	+	1.75	7.00E-06	3.96E-04

organic cyclic compound binding (GO:0097159)	229	66	38.38	+	1.72	1.49E-04	6.04E-03
heterocyclic compound binding (GO:1901363)	229	66	38.38	+	1.72	1.49E-04	5.28E-03
catalytic activity (GO:0003824)	842	241	141.13	+	1.71	1.53E-15	4.33E-13
hydrolase activity (GO:0016787)	228	60	38.22	+	1.57	2.34E-03	4.41E-02
molecular_function (GO:0003674)	1283	325	215.05	+	1.51	3.79E-15	5.37E-13
Unclassified (UNCLASSIFIED)	3108	411	520.95	-	0.79	3.79E-15	3.58E-13
transporter activity (GO:0005215)	236	18	39.56	-	0.46	3.23E-04	1.02E-02
transmembrane transporter activity (GO:0022857)	231	16	38.72	-	0.41	1.20E-04	5.68E-03

Analysis Type:	PANTHER Overrepresentation Test (Released 20200728)
Annotation Version and Release Date:	PANTHER version 15.0 Released 2020-02-14
Analyzed List:	protein.txt (Homo sapiens)
Reference List:	Homo sapiens (all genes in database)
Test Type:	FISHER
Correction:	FDR

PANTHER GO-Slim Biological Process	Homo sap	protein.txt	protein.txt	protein.txt	protein.txt	protein.txt	protein.txt
pre-replicative complex assembly involved in nuclear cell cycle DNA replication (GO:0006267)	8	4	0.15	+	26.46	5.13E-05	1.07E-03
double-strand break repair via break-induced replication (GO:0000727)	12	4	0.23	+	17.64	1.78E-04	3.08E-03

DNA unwinding involved in DNA replication (GO:0006268)	10	3	0.19	+	15.88	1.58E-03	2.00E-02
mitochondrial RNA metabolic process (GO:0000959)	10	3	0.19	+	15.88	1.58E-03	1.98E-02
tricarboxylic acid cycle (GO:0006099)	17	5	0.32	+	15.57	4.34E-05	9.24E-04
ATP biosynthetic process (GO:0006754)	38	11	0.72	+	15.32	1.20E-09	7.09E-08
ATP synthesis coupled proton transport (GO:0015986)	38	11	0.72	+	15.32	1.20E-09	6.90E-08
energy coupled proton transport, down electrochemical gradient (GO:0015985)	38	11	0.72	+	15.32	1.20E-09	6.71E-08
purine nucleoside triphosphate biosynthetic process (GO:0009145)	40	11	0.76	+	14.55	1.90E-09	9.13E-08

purine ribonucleoside triphosphate biosynthetic process (GO:0009206)	40	11	0.76	+	14.55	1.90E-09	8.92E-08
purine ribonucleoside triphosphate metabolic process (GO:0009205)	40	11	0.76	+	14.55	1.90E-09	8.72E-08
mitochondrial ATP synthesis coupled electron transport (GO:0042775)	22	6	0.42	+	14.43	1.04E-05	2.57E-04
DNA strand elongation involved in DNA replication (GO:0006271)	11	3	0.21	+	14.43	1.98E-03	2.46E-02
purine nucleoside triphosphate metabolic process (GO:0009144)	41	11	0.77	+	14.2	2.37E-09	1.06E-07
nucleoside triphosphate biosynthetic process (GO:0009142)	45	12	0.85	+	14.11	4.67E-10	3.44E-08

ribonucleoside triphosphate metabolic process (GO:0009199)	42	11	0.79	+	13.86	2.94E-09	1.22E-07
ribonucleoside triphosphate biosynthetic process (GO:0009201)	42	11	0.79	+	13.86	2.94E-09	1.19E-07
ATP synthesis coupled electron transport (GO:0042773)	23	6	0.43	+	13.81	1.30E-05	3.11E-04
nucleoside triphosphate metabolic process (GO:0009141)	51	13	0.96	+	13.49	1.41E-10	1.32E-08
cell cycle DNA replication (GO:0044786)	16	4	0.3	+	13.23	4.46E-04	6.82E-03
nuclear DNA replication (GO:0033260)	16	4	0.3	+	13.23	4.46E-04	6.77E-03
proton transmembrane transport (GO:1902600)	49	12	0.93	+	12.96	1.08E-09	6.53E-08
oxidative phosphorylation (GO:0006119)	29	7	0.55	+	12.77	3.75E-06	1.02E-04

DNA replication initiation (GO:0006270)	17	4	0.32	+	12.45	5.43E-04	8.12E-03
respiratory electron transport chain (GO:0022904)	31	7	0.59	+	11.95	5.50E-06	1.44E-04
electron transport chain (GO:0022900)	31	7	0.59	+	11.95	5.50E-06	1.42E-04
aerobic respiration (GO:0009060)	38	8	0.72	+	11.14	1.84E-06	5.34E-05
cellular respiration (GO:0045333)	57	12	1.08	+	11.14	4.77E-09	1.86E-07
ATP metabolic process (GO:0046034)	56	11	1.06	+	10.4	3.93E-08	1.38E-06
mitochondrial gene expression (GO:0140053)	26	5	0.49	+	10.18	2.44E-04	4.13E-03
purine ribonucleotide biosynthetic process (GO:0009152)	74	14	1.4	+	10.01	8.38E-10	5.41E-08
purine nucleotide biosynthetic process (GO:0006164)	80	15	1.51	+	9.92	2.32E-10	2.00E-08

fatty acid catabolic process (GO:0009062)	27	5	0.51	+	9.8	2.85E-04	4.75E-03
ribonucleotide biosynthetic process (GO:0009260)	78	14	1.47	+	9.5	1.54E-09	7.73E-08
purine-containing compound biosynthetic process (GO:0072522)	84	15	1.59	+	9.45	4.23E-10	3.36E-08
energy derivation by oxidation of organic compounds (GO:0015980)	68	12	1.28	+	9.34	2.71E-08	9.65E-07
ribose phosphate biosynthetic process (GO:0046390)	83	14	1.57	+	8.93	3.13E-09	1.24E-07
nucleotide biosynthetic process (GO:0009165)	103	16	1.95	+	8.22	6.84E-10	4.87E-08
nucleoside phosphate biosynthetic process (GO:1901293)	104	16	1.97	+	8.14	7.76E-10	5.34E-08

generation of precursor metabolites and energy (GO:0006091)	103	15	1.95	+	7.71	5.16E-09	1.94E-07
cellular amino acid metabolic process (GO:0006520)	131	19	2.48	+	7.68	4.89E-11	4.81E-09
translational elongation (GO:0006414)	225	32	4.25	+	7.53	1.66E-17	6.84E-15
translation (GO:0006412)	225	32	4.25	+	7.53	1.66E-17	5.70E-15
peptide biosynthetic process (GO:0043043)	228	32	4.31	+	7.43	2.34E-17	6.90E-15
purine nucleotide metabolic process (GO:0006163)	133	18	2.51	+	7.16	4.37E-10	3.34E-08
purine ribonucleotide metabolic process (GO:0009150)	126	17	2.38	+	7.14	1.40E-09	7.60E-08
amide biosynthetic process (GO:0043604)	259	34	4.89	+	6.95	1.43E-17	7.38E-15
ribonucleotide metabolic process (GO:0009259)	131	17	2.48	+	6.87	2.39E-09	1.05E-07

regulation of mRNA splicing, via spliceosome (GO:0048024)	56	7	1.06	+	6.62	1.62E-04	2.93E-03
acyl-CoA metabolic process (GO:0006637)	32	4	0.6	+	6.62	4.29E-03	4.74E-02
purine-containing compound metabolic process (GO:0072521)	145	18	2.74	+	6.57	1.53E-09	7.90E-08
ribose phosphate metabolic process (GO:0019693)	138	17	2.61	+	6.52	4.87E-09	1.86E-07
regulation of RNA splicing (GO:0043484)	65	8	1.23	+	6.51	6.12E-05	1.25E-03
carboxylic acid catabolic process (GO:0046395)	74	9	1.4	+	6.44	2.32E-05	5.32E-04
organic acid catabolic process (GO:0016054)	74	9	1.4	+	6.44	2.32E-05	5.26E-04
tRNA metabolic process (GO:0006399)	107	13	2.02	+	6.43	3.68E-07	1.13E-05

nucleoside phosphate metabolic process (GO:0006753)	165	20	3.12	+	6.41	2.74E-10	2.26E-08
peptide metabolic process (GO:0006518)	292	35	5.52	+	6.34	6.11E-17	1.40E-14
regulation of mRNA processing (GO:0050684)	67	8	1.27	+	6.32	7.44E-05	1.43E-03
nucleotide metabolic process (GO:0009117)	160	19	3.02	+	6.28	1.05E-09	6.58E-08
cytoplasmic translation (GO:0002181)	52	6	0.98	+	6.11	7.03E-04	1.01E-02
actin polymerization or depolymerization (GO:0008154)	80	9	1.51	+	5.95	4.07E-05	8.84E-04
organophosphate biosynthetic process (GO:0090407)	169	19	3.19	+	5.95	2.41E-09	1.04E-07
regulation of actin filament length (GO:0030832)	74	8	1.4	+	5.72	1.40E-04	2.59E-03

regulation of actin polymerization or depolymerization (GO:0008064)	74	8	1.4	+	5.72	1.40E-04	2.56E-03
oxidation-reduction process (GO:0055114)	185	20	3.5	+	5.72	1.69E-09	8.32E-08
cellular amide metabolic process (GO:0043603)	370	40	6.99	+	5.72	8.81E-18	6.06E-15
nucleobase-containing small molecule metabolic process (GO:0055086)	197	21	3.72	+	5.64	8.33E-10	5.55E-08
drug metabolic process (GO:0017144)	165	17	3.12	+	5.45	5.44E-08	1.87E-06
positive regulation of supramolecular fiber organization (GO:1902905)	49	5	0.93	+	5.4	3.21E-03	3.66E-02
positive regulation of cytoskeleton organization (GO:0051495)	51	5	0.96	+	5.19	3.76E-03	4.24E-02

regulation of actin filament organization (GO:0110053)	82	8	1.55	+	5.16	2.69E-04	4.51E-03
regulation of actin filament polymerization (GO:0030833)	72	7	1.36	+	5.15	6.59E-04	9.64E-03
carboxylic acid metabolic process (GO:0019752)	315	30	5.95	+	5.04	2.58E-12	3.55E-10
actin filament polymerization (GO:0030041)	75	7	1.42	+	4.94	8.24E-04	1.16E-02
regulation of mRNA metabolic process (GO:1903311)	87	8	1.64	+	4.87	3.89E-04	6.12E-03
fatty acid metabolic process (GO:0006631)	98	9	1.85	+	4.86	1.71E-04	3.00E-03
oxoacid metabolic process (GO:0043436)	327	30	6.18	+	4.86	6.14E-12	7.92E-10
regulation of protein polymerization (GO:0032271)	77	7	1.45	+	4.81	9.51E-04	1.31E-02
organic acid metabolic process (GO:0006082)	331	30	6.25	+	4.8	8.13E-12	9.88E-10

small molecule metabolic process (GO:0044281)	564	50	10.66	+	4.69	1.22E-18	2.53E-15
small molecule catabolic process (GO:0044282)	113	10	2.14	+	4.68	1.00E-04	1.88E-03
regulation of supramolecular fiber organization (GO:1902903)	93	8	1.76	+	4.55	5.87E-04	8.65E-03
alpha-amino acid metabolic process (GO:1901605)	82	7	1.55	+	4.52	1.34E-03	1.78E-02
organonitrogen compound biosynthetic process (GO:1901566)	602	51	11.38	+	4.48	3.18E-18	3.28E-15
mitochondrial transport (GO:0006839)	72	6	1.36	+	4.41	3.27E-03	3.71E-02
regulation of protein complex assembly (GO:0043254)	97	8	1.83	+	4.36	7.60E-04	1.09E-02
mRNA splicing, via spliceosome (GO:0000398)	156	12	2.95	+	4.07	7.36E-05	1.46E-03

RNA splicing, via transesterification reactions with bulged adenosine as nucleophile (GO:0000377)	156	12	2.95	+	4.07	7.36E-05	1.45E-03
RNA splicing, via transesterification reactions (GO:0000375)	156	12	2.95	+	4.07	7.36E-05	1.43E-03
protein polymerization (GO:0051258)	91	7	1.72	+	4.07	2.33E-03	2.81E-02
protein-DNA complex assembly (GO:0065004)	91	7	1.72	+	4.07	2.33E-03	2.80E-02
ncRNA metabolic process (GO:0034660)	260	20	4.91	+	4.07	3.16E-07	9.89E-06
regulation of cellular component size (GO:0032535)	107	8	2.02	+	3.96	1.37E-03	1.82E-02
cellular protein-containing complex assembly (GO:0034622)	391	29	7.39	+	3.93	1.46E-09	7.75E-08

regulation of actin cytoskeleton organization (GO:0032956)	108	8	2.04	+	3.92	1.45E-03	1.89E-02
regulation of actin filament-based process (GO:0032970)	109	8	2.06	+	3.88	1.53E-03	1.97E-02
regulation of cytoskeleton organization (GO:0051493)	150	11	2.83	+	3.88	2.16E-04	3.71E-03
protein folding (GO:0006457)	96	7	1.81	+	3.86	3.09E-03	3.54E-02
RNA splicing (GO:0008380)	179	13	3.38	+	3.84	6.46E-05	1.31E-03
organophosphate metabolic process (GO:0019637)	310	22	5.86	+	3.76	2.97E-07	9.58E-06
ribonucleoprotein complex assembly (GO:0022618)	113	8	2.14	+	3.75	1.90E-03	2.38E-02
protein-containing complex assembly (GO:0065003)	420	29	7.94	+	3.65	6.63E-09	2.45E-07
protein-DNA complex subunit organization (GO:0071824)	116	8	2.19	+	3.65	2.22E-03	2.71E-02

ribonucleoprotein complex subunit organization (GO:0071826)	118	8	2.23	+	3.59	2.45E-03	2.92E-02
regulation of anatomical structure size (GO:0090066)	119	8	2.25	+	3.56	2.57E-03	3.02E-02
protein-containing complex subunit organization (GO:0043933)	511	33	9.66	+	3.42	2.80E-09	1.18E-07
monocarboxylic acid metabolic process (GO:0032787)	155	10	2.93	+	3.41	1.05E-03	1.44E-02
mRNA processing (GO:0006397)	206	13	3.89	+	3.34	2.41E-04	4.10E-03
regulation of cellular component biogenesis (GO:0044087)	144	9	2.72	+	3.31	2.27E-03	2.75E-02
ribosome biogenesis (GO:0042254)	177	11	3.34	+	3.29	8.03E-04	1.14E-02
actin filament organization (GO:0007015)	161	10	3.04	+	3.29	1.38E-03	1.81E-02

small molecule biosynthetic process (GO:0044283)	162	10	3.06	+	3.27	1.44E-03	1.88E-02
carbohydrate derivative biosynthetic process (GO:1901137)	244	15	4.61	+	3.25	1.07E-04	2.00E-03
monovalent inorganic cation transport (GO:0015672)	202	12	3.82	+	3.14	6.87E-04	9.99E-03
ribonucleoprotein complex biogenesis (GO:0022613)	240	14	4.54	+	3.09	3.01E-04	4.82E-03
mRNA metabolic process (GO:0016071)	269	15	5.08	+	2.95	2.93E-04	4.83E-03
supramolecular fiber organization (GO:0097435)	238	13	4.5	+	2.89	8.74E-04	1.21E-02
RNA processing (GO:0006396)	433	23	8.18	+	2.81	1.61E-05	3.77E-04
organonitrogen compound catabolic process (GO:1901565)	439	23	8.3	+	2.77	1.98E-05	4.58E-04

carbohydrate derivative metabolic process (GO:1901135)	377	19	7.12	+	2.67	1.67E-04	3.00E-03
organic substance catabolic process (GO:1901575)	678	34	12.81	+	2.65	5.23E-07	1.59E-05
cellular macromolecule catabolic process (GO:0044265)	410	20	7.75	+	2.58	1.71E-04	3.02E-03
proteolysis involved in cellular protein catabolic process (GO:0051603)	308	15	5.82	+	2.58	1.10E-03	1.50E-02
cellular protein catabolic process (GO:0044257)	310	15	5.86	+	2.56	1.17E-03	1.58E-02
protein catabolic process (GO:0030163)	333	16	6.29	+	2.54	8.74E-04	1.22E-02
macromolecule catabolic process (GO:0009057)	442	21	8.35	+	2.51	1.67E-04	2.98E-03
inorganic cation transmembrane transport (GO:0098662)	274	13	5.18	+	2.51	2.87E-03	3.31E-02

cellular catabolic process (GO:0044248)	723	34	13.66	+	2.49	2.60E-06	7.25E-05
cation transmembrane transport (GO:0098655)	302	14	5.71	+	2.45	2.46E-03	2.92E-02
inorganic ion transmembrane transport (GO:0098660)	305	14	5.76	+	2.43	2.68E-03	3.13E-02
catabolic process (GO:0009056)	828	35	15.65	+	2.24	1.47E-05	3.49E-04
cellular component biogenesis (GO:0044085)	937	39	17.71	+	2.2	7.55E-06	1.88E-04
cellular component assembly (GO:0022607)	806	33	15.23	+	2.17	6.05E-05	1.25E-03
organonitrogen compound metabolic process (GO:1901564)	2386	97	45.09	+	2.15	5.48E-13	1.03E-10
cellular nitrogen compound metabolic process (GO:0034641)	2406	96	45.46	+	2.11	2.24E-12	3.56E-10

cellular nitrogen compound biosynthetic process (GO:0044271)	1633	62	30.86	+	2.01	2.52E-07	8.25E-06
cellular protein metabolic process (GO:0044267)	1679	62	31.73	+	1.95	6.14E-07	1.84E-05
cellular biosynthetic process (GO:0044249)	1957	71	36.98	+	1.92	1.43E-07	4.84E-06
cellular metabolic process (GO:0044237)	4268	154	80.65	+	1.91	6.08E-17	1.57E-14
organic substance biosynthetic process (GO:1901576)	1976	71	37.34	+	1.9	2.46E-07	8.18E-06
biosynthetic process (GO:0009058)	1997	71	37.74	+	1.88	3.03E-07	9.63E-06
metabolic process (GO:0008152)	4633	156	87.55	+	1.78	1.79E-14	3.69E-12
heterocycle metabolic process (GO:0046483)	2151	72	40.65	+	1.77	2.55E-06	7.21E-05
primary metabolic process (GO:0044238)	4250	142	80.31	+	1.77	1.49E-12	2.57E-10

nucleobase-containing compound metabolic process (GO:0006139)	2110	70	39.87	+	1.76	4.83E-06	1.28E-04
nitrogen compound metabolic process (GO:0006807)	4049	134	76.51	+	1.75	1.59E-11	1.64E-09
organic cyclic compound metabolic process (GO:1901360)	2207	73	41.7	+	1.75	3.32E-06	9.12E-05
cellular aromatic compound metabolic process (GO:0006725)	2162	71	40.85	+	1.74	6.07E-06	1.55E-04
organic substance metabolic process (GO:0071704)	4460	146	84.28	+	1.73	2.42E-12	3.57E-10
gene expression (GO:0010467)	1925	62	36.37	+	1.7	5.00E-05	1.05E-03
protein metabolic process (GO:0019538)	1981	62	37.43	+	1.66	9.58E-05	1.81E-03
cellular macromolecule metabolic process (GO:0044260)	3069	84	57.99	+	1.45	5.61E-04	8.34E-03

macromolecule metabolic process (GO:0043170)	3732	99	70.52	+	1.4	4.32E-04	6.65E-03
cellular process (GO:0009987)	7481	197	141.36	+	1.39	1.63E-08	5.89E-07
biological_process (GO:0008150)	8857	209	167.36	+	1.25	3.00E-05	6.73E-04
Unclassified (UNCLASSIFIED)	11994	185	226.64	-	0.82	3.00E-05	6.66E-04
biological regulation (GO:0065007)	4469	52	84.45	-	0.62	3.85E-05	8.45E-04
regulation of biological process (GO:0050789)	4002	42	75.62	-	0.56	6.96E-06	1.75E-04
regulation of cellular process (GO:0050794)	3779	37	71.41	-	0.52	2.18E-06	6.25E-05
regulation of macromolecule biosynthetic process (GO:0010556)	1194	10	22.56	-	0.44	4.06E-03	4.55E-02
regulation of cellular biosynthetic process (GO:0031326)	1205	10	22.77	-	0.44	4.11E-03	4.58E-02

regulation of biosynthetic process (GO:0009889)	1211	10	22.88	-	0.44	4.16E-03	4.62E-02
cellular response to stimulus (GO:0051716)	2360	19	44.59	-	0.43	1.13E-05	2.75E-04
response to stimulus (GO:0050896)	2802	22	52.95	-	0.42	8.74E-07	2.58E-05
RNA biosynthetic process (GO:0032774)	1139	8	21.52	-	0.37	1.46E-03	1.88E-02
multicellular organismal process (GO:0032501)	1174	8	22.18	-	0.36	7.93E-04	1.13E-02
nucleic acid-templated transcription (GO:0097659)	1132	7	21.39	-	0.33	4.26E-04	6.66E-03
transcription, DNA-templated (GO:0006351)	1132	7	21.39	-	0.33	4.26E-04	6.61E-03
regulation of transcription, DNA-templated (GO:0006355)	1081	6	20.43	-	0.29	2.97E-04	4.87E-03

regulation of nucleic acid-templated transcription (GO:1903506)	1081	6	20.43	-	0.29	2.97E-04	4.83E-03
regulation of RNA biosynthetic process (GO:2001141)	1081	6	20.43	-	0.29	2.97E-04	4.79E-03
regulation of transcription by RNA polymerase II (GO:0006357)	772	4	14.59	-	0.27	2.48E-03	2.92E-02
transcription by RNA polymerase II (GO:0006366)	808	4	15.27	-	0.26	1.26E-03	1.69E-02
regulation of cell communication (GO:0010646)	685	3	12.94	-	0.23	2.19E-03	2.70E-02
regulation of signaling (GO:0023051)	687	3	12.98	-	0.23	2.20E-03	2.71E-02
regulation of response to stimulus (GO:0048583)	893	3	16.87	-	0.18	6.85E-05	1.37E-03
signal transduction (GO:0007165)	1887	5	35.66	-	0.14	1.49E-10	1.34E-08

intracellular signal transduction (GO:0035556)	800	2	15.12	-	0.13	7.52E-05	1.44E-03
signaling (GO:0023052)	2027	5	38.3	-	0.13	9.11E-12	1.04E-09
cell communication (GO:0007154)	2031	5	38.38	-	0.13	9.13E-12	9.92E-10
positive regulation of response to stimulus (GO:0048584)	469	1	8.86	-	0.11	2.72E-03	3.15E-02
cell surface receptor signaling pathway (GO:0007166)	829	1	15.66	-	0.06	4.53E-06	1.22E-04
cell-cell signaling (GO:0007267)	381	0	7.2	-	< 0.01	1.57E-03	2.00E-02
immune system process (GO:0002376)	556	0	10.51	-	< 0.01	4.33E-05	9.30E-04
G protein-coupled receptor signaling pathway (GO:0007186)	453	0	8.56	-	< 0.01	3.13E-04	4.97E-03
immune response (GO:0006955)	449	0	8.48	-	< 0.01	5.05E-04	7.61E-03

(FDR)

Analysis Type: PANTHER Overrepresentation Test (Released 20200728)

Annotation Version and Release Date: PANTHER version 15.0 Released 2020-02-14

Analyzed List: protein.txt (Homo sapiens)

Reference List: Homo sapiens (all genes in database)

Test Type: FISHER

Correction: FDR

PANTHER GO-Slim Cellular Component	Homo sap	protein.txt	protein.txt	protein.txt	protein.txt	protein.txt	protein.txt (FDR)
mitochondrial respiratory chain complex III (GO:0005750)	5	2	0.09	+	21.17	6.77E-03	4.14E-02
proteasome core complex, alpha-subunit complex (GO:0019773)	8	3	0.15	+	19.85	9.35E-04	7.84E-03
mitochondrial proton-transporting ATP synthase complex (GO:0005753)	11	4	0.21	+	19.24	1.35E-04	1.50E-03

mitochondrial small ribosomal subunit (GO:0005763)	12	4	0.23	+	17.64	1.78E-04	1.81E-03
proton-transporting ATP synthase complex (GO:0045259)	12	4	0.23	+	17.64	1.78E-04	1.78E-03
Arp2/3 protein complex (GO:0005885)	10	3	0.19	+	15.88	1.58E-03	1.28E-02
chaperonin-containing T-complex (GO:0005832)	12	3	0.23	+	13.23	2.44E-03	1.84E-02
small ribosomal subunit (GO:0015935)	55	12	1.04	+	11.55	3.35E-09	8.72E-08
proteasome accessory complex (GO:0022624)	16	3	0.3	+	9.92	4.92E-03	3.28E-02
proteasome regulatory particle (GO:0005838)	16	3	0.3	+	9.92	4.92E-03	3.24E-02
cytosolic large ribosomal subunit (GO:0022625)	54	10	1.02	+	9.8	2.67E-07	4.95E-06
cytosolic ribosome (GO:0022626)	94	17	1.78	+	9.57	2.40E-11	1.13E-09

cytosolic small ribosomal subunit (GO:0022627)	40	7	0.76	+	9.26	2.37E-05	3.09E-04
ribosomal subunit (GO:0044391)	132	22	2.49	+	8.82	1.17E-13	8.67E-12
ribosome (GO:0005840)	140	23	2.65	+	8.69	4.19E-14	3.63E-12
mitochondrial protein complex (GO:0098798)	129	21	2.44	+	8.62	6.39E-13	4.15E-11
integral component of mitochondrial inner membrane (GO:0031305)	25	4	0.47	+	8.47	1.92E-03	1.53E-02
intrinsic component of mitochondrial inner membrane (GO:0031304)	25	4	0.47	+	8.47	1.92E-03	1.51E-02
inner mitochondrial membrane protein complex (GO:0098800)	75	12	1.42	+	8.47	7.10E-08	1.48E-06
mitochondrial respiratory chain complex I (GO:0005747)	25	4	0.47	+	8.47	1.92E-03	1.49E-02

oxidoreductase complex (GO:1990204)	45	7	0.85	+	8.23	4.66E-05	5.77E-04
mitochondrial respirasome (GO:0005746)	45	7	0.85	+	8.23	4.66E-05	5.64E-04
endopeptidase complex (GO:1905369)	39	6	0.74	+	8.14	1.73E-04	1.88E-03
cortical actin cytoskeleton (GO:0030864)	39	6	0.74	+	8.14	1.73E-04	1.84E-03
proton-transporting two-sector ATPase complex (GO:0016469)	26	4	0.49	+	8.14	2.18E-03	1.67E-02
proteasome complex (GO:0000502)	39	6	0.74	+	8.14	1.73E-04	1.80E-03
respiratory chain complex I (GO:0045271)	27	4	0.51	+	7.84	2.47E-03	1.83E-02
NADH dehydrogenase complex (GO:0030964)	28	4	0.53	+	7.56	2.78E-03	2.01E-02
respirasome (GO:0070469)	50	7	0.94	+	7.41	8.51E-05	1.01E-03
cortical cytoskeleton (GO:0030863)	43	6	0.81	+	7.38	2.79E-04	2.74E-03

mitochondrial inner membrane (GO:0005743)	129	18	2.44	+	7.38	2.80E-10	8.56E-09
respiratory chain complex (GO:0098803)	45	6	0.85	+	7.06	3.49E-04	3.24E-03
mitochondrial membrane part (GO:0044455)	114	15	2.15	+	6.96	1.77E-08	4.19E-07
large ribosomal subunit (GO:0015934)	77	10	1.45	+	6.87	4.82E-06	6.97E-05
cytosolic part (GO:0044445)	156	20	2.95	+	6.78	1.11E-10	3.85E-09
organelle inner membrane (GO:0019866)	143	18	2.7	+	6.66	1.25E-09	3.43E-08
MCM core complex (GO:0097373)	33	4	0.62	+	6.41	4.75E-03	3.25E-02
MCM complex (GO:0042555)	33	4	0.62	+	6.41	4.75E-03	3.20E-02
organellar ribosome (GO:0000313)	35	4	0.66	+	6.05	5.74E-03	3.64E-02
integral component of mitochondrial membrane (GO:0032592)	35	4	0.66	+	6.05	5.74E-03	3.60E-02

mitochondrial ribosome (GO:0005761)	35	4	0.66	+	6.05	5.74E-03	3.55E-02
mitochondrial matrix (GO:0005759)	62	7	1.17	+	5.97	2.87E-04	2.71E-03
mitochondrion (GO:0005739)	470	53	8.88	+	5.97	4.35E-24	1.13E-21
intrinsic component of mitochondrial membrane (GO:0098573)	37	4	0.7	+	5.72	6.87E-03	4.15E-02
peptidase complex (GO:1905368)	56	6	1.06	+	5.67	1.00E-03	8.28E-03
mitochondrial part (GO:0044429)	249	25	4.71	+	5.31	6.45E-11	2.40E-09
mitochondrial membrane (GO:0031966)	180	18	3.4	+	5.29	3.36E-08	7.59E-07
mitochondrial envelope (GO:0005740)	193	18	3.65	+	4.94	8.91E-08	1.78E-06
cell cortex part (GO:0044448)	91	8	1.72	+	4.65	5.13E-04	4.60E-03
spliceosomal snRNP complex (GO:0097525)	76	6	1.44	+	4.18	4.19E-03	2.95E-02

ribonucleoprotein complex (GO:1990904)	457	36	8.64	+	4.17	2.79E-12	1.61E-10
small nuclear ribonucleoprotein complex (GO:0030532)	77	6	1.45	+	4.12	4.45E-03	3.09E-02
Sm-like protein family complex (GO:0120114)	80	6	1.51	+	3.97	5.30E-03	3.40E-02
cell cortex (GO:0005938)	122	9	2.31	+	3.9	7.67E-04	6.54E-03
envelope (GO:0031975)	296	20	5.59	+	3.58	2.10E-06	3.41E-05
organelle envelope (GO:0031967)	296	20	5.59	+	3.58	2.10E-06	3.30E-05
cytosol (GO:0005829)	710	45	13.42	+	3.35	5.40E-12	2.81E-10
actin cytoskeleton (GO:0015629)	163	9	3.08	+	2.92	4.92E-03	3.20E-02
cytoplasmic part (GO:0044444)	2913	125	55.04	+	2.27	8.10E-19	8.43E-17
cytoplasm (GO:0005737)	4104	168	77.55	+	2.17	1.33E-24	6.89E-22
intracellular non-membrane-bounded organelle (GO:0043232)	1284	49	24.26	+	2.02	4.62E-06	7.07E-05

non-membrane-bounded organelle (GO:0043228)	1284	49	24.26	+	2.02	4.62E-06	6.87E-05
protein-containing complex (GO:0032991)	2713	94	51.26	+	1.83	7.26E-09	1.80E-07
intracellular organelle part (GO:0044446)	2610	89	49.32	+	1.8	4.15E-08	9.00E-07
organelle part (GO:0044422)	2695	90	50.92	+	1.77	9.54E-08	1.84E-06
intracellular (GO:0005622)	6661	215	125.87	+	1.71	6.14E-20	1.06E-17
intracellular part (GO:0044424)	6630	213	125.28	+	1.7	2.62E-19	3.41E-17
intracellular organelle (GO:0043229)	5308	159	100.3	+	1.59	1.55E-10	5.04E-09
organelle (GO:0043226)	5421	159	102.44	+	1.55	9.13E-10	2.64E-08
intracellular membrane-bounded organelle (GO:0043231)	4649	127	87.85	+	1.45	7.78E-06	1.09E-04
membrane-bounded organelle (GO:0043227)	4717	127	89.13	+	1.42	1.53E-05	2.10E-04
cell part (GO:0044464)	8223	221	155.38	+	1.42	4.13E-11	1.79E-09

cell (GO:0005623)	8223	221	155.38	+	1.42	4.13E-11	1.65E-09
cellular_component (GO:0005575)	9090	223	171.76	+	1.3	3.42E-07	6.13E-06
Unclassified (UNCLASSIFIED)	11761	171	222.24	-	0.77	3.42E-07	5.92E-06
membrane (GO:0016020)	3445	44	65.1	-	0.68	3.85E-03	2.74E-02
cell periphery (GO:0071944)	3175	31	59.99	-	0.52	1.80E-05	2.40E-04
plasma membrane (GO:0005886)	3105	26	58.67	-	0.44	6.10E-07	1.02E-05
extracellular space (GO:0005615)	850	5	16.06	-	0.31	2.59E-03	1.90E-02
plasma membrane part (GO:0044459)	1404	8	26.53	-	0.3	3.58E-05	4.54E-04
extracellular region (GO:0005576)	937	5	17.71	-	0.28	7.29E-04	6.42E-03
extracellular region part (GO:0044421)	937	5	17.71	-	0.28	7.29E-04	6.31E-03
integral component of membrane (GO:0016021)	986	5	18.63	-	0.27	3.88E-04	3.54E-03
intrinsic component of membrane (GO:0031224)	1012	5	19.12	-	0.26	2.85E-04	2.74E-03

integral component of plasma membrane (GO:0005887)	652	1	12.32	-	0.08	9.40E-05	1.09E-03
intrinsic component of plasma membrane (GO:0031226)	660	1	12.47	-	0.08	9.57E-05	1.08E-03

Analysis Type:	PANTHER Overrepresentation Test (Released 20200728)
Annotation Version and Release Date:	PANTHER version 15.0 Released 2020-02-14
Analyzed List:	protein.txt (Homo sapiens)
Reference List:	Homo sapiens (all genes in database)
Test Type:	FISHER
Correction:	FDR

PANTHER GO-Slim Molecular Function	Homo sap	protein.txt	protein.txt	protein.txt	protein.txt	protein.txt	protein.txt (FDR)
proton-transporting ATP synthase activity, rotational mechanism (GO:0046933)	9	4	0.17	+	23.52	7.30E-05	1.76E-03
pre-mRNA binding (GO:0036002)	14	4	0.26	+	15.12	2.90E-04	5.51E-03
magnesium ion binding (GO:0000287)	11	3	0.21	+	14.43	1.98E-03	2.24E-02
3'-5' DNA helicase activity (GO:0043138)	17	4	0.32	+	12.45	5.43E-04	8.25E-03

DNA replication origin binding (GO:0003688)	18	4	0.34	+	11.76	6.54E-04	9.66E-03
electron transfer activity (GO:0009055)	24	5	0.45	+	11.03	1.76E-04	3.74E-03
structural constituent of ribosome (GO:0003735)	119	23	2.25	+	10.23	1.94E-15	5.17E-13
hydro-lyase activity (GO:0016836)	28	5	0.53	+	9.45	3.31E-04	5.87E-03
carbon-oxygen lyase activity (GO:0016835)	28	5	0.53	+	9.45	3.31E-04	5.68E-03
endoribonuclease activity (GO:0004521)	34	6	0.64	+	9.34	8.82E-05	2.04E-03
ribonucleoprotein complex binding (GO:0043021)	42	7	0.79	+	8.82	3.14E-05	8.35E-04
single-stranded RNA binding (GO:0003727)	31	5	0.59	+	8.54	5.02E-04	7.86E-03
ligase activity (GO:0016874)	93	15	1.76	+	8.54	1.48E-09	9.84E-08
catalytic activity, acting on a tRNA (GO:0140101)	86	13	1.63	+	8	3.69E-08	1.96E-06

structural molecule activity (GO:0005198)	215	25	4.06	+	6.15	3.54E-12	3.14E-10
acetyltransferase activity (GO:0016407)	52	6	0.98	+	6.11	7.03E-04	1.01E-02
endonuclease activity (GO:0004519)	57	6	1.08	+	5.57	1.09E-03	1.38E-02
catalytic activity, acting on RNA (GO:0140098)	210	22	3.97	+	5.54	4.43E-10	3.37E-08
ribonuclease activity (GO:0004540)	67	7	1.27	+	5.53	4.43E-04	7.13E-03
transition metal ion binding (GO:0046914)	83	8	1.57	+	5.1	2.90E-04	5.71E-03
oxidoreductase activity, acting on the CH-OH group of donors, NAD or NADP as acceptor (GO:0016616)	64	6	1.21	+	4.96	1.89E-03	2.19E-02
actin filament binding (GO:0051015)	86	8	1.63	+	4.92	3.62E-04	6.01E-03
translation regulator activity (GO:0045182)	71	6	1.34	+	4.47	3.07E-03	3.26E-02

lyase activity (GO:0016829)	86	7	1.63	+	4.31	1.73E-03	2.04E-02
RNA binding (GO:0003723)	516	40	9.75	+	4.1	2.57E-13	2.73E-11
actin binding (GO:0003779)	131	10	2.48	+	4.04	3.07E-04	5.64E-03
oxidoreductase activity (GO:0016491)	388	27	7.33	+	3.68	1.92E-08	1.14E-06
ATPase activity, coupled (GO:0042623)	117	8	2.21	+	3.62	2.33E-03	2.58E-02
nuclease activity (GO:0004518)	120	8	2.27	+	3.53	2.70E-03	2.93E-02
protein-containing complex binding (GO:0044877)	274	18	5.18	+	3.48	9.72E-06	2.72E-04
ATPase activity (GO:0016887)	262	16	4.95	+	3.23	6.87E-05	1.74E-03
metal ion binding (GO:0046872)	224	11	4.23	+	2.6	4.61E-03	4.81E-02
cytoskeletal protein binding (GO:0008092)	345	16	6.52	+	2.45	1.24E-03	1.54E-02
pyrophosphatase activity (GO:0016462)	565	23	10.68	+	2.15	8.57E-04	1.20E-02

hydrolase activity, acting on acid anhydrides, in phosphorus-containing anhydrides (GO:0016818)	567	23	10.71	+	2.15	8.82E-04	1.20E-02
hydrolase activity, acting on acid anhydrides (GO:0016817)	567	23	10.71	+	2.15	8.82E-04	1.17E-02
catalytic activity (GO:0003824)	3598	137	67.99	+	2.02	1.37E-16	7.30E-14
nucleic acid binding (GO:0003676)	1325	50	25.04	+	2	6.12E-06	2.32E-04
heterocyclic compound binding (GO:1901363)	1646	59	31.1	+	1.9	3.30E-06	1.46E-04
organic cyclic compound binding (GO:0097159)	1677	59	31.69	+	1.86	6.28E-06	2.23E-04
hydrolase activity (GO:0016787)	1584	51	29.93	+	1.7	2.55E-04	5.22E-03
molecular_function (GO:0003674)	8266	230	156.19	+	1.47	1.35E-13	1.80E-11
binding (GO:0005488)	4589	115	86.71	+	1.33	9.26E-04	1.20E-02

Unclassified (UNCLASSIFIED)	12585	164	237.81	-	0.69	1.35E-13	2.39E-11
signaling receptor binding (GO:0005102)	629	1	11.89	-	0.08	1.37E-04	3.04E-03
transcription regulator activity (GO:0140110)	816	1	15.42	-	0.06	7.02E-06	2.33E-04
molecular transducer activity (GO:0060089)	820	1	15.49	-	0.06	4.44E-06	1.82E-04
transmembrane signaling receptor activity (GO:0004888)	640	0	12.09	-	< 0.01	8.83E-06	2.76E-04
DNA-binding transcription factor activity, RNA polymerase II-specific (GO:0000981)	373	0	7.05	-	< 0.01	1.52E-03	1.84E-02
DNA-binding transcription factor activity (GO:0003700)	647	0	12.23	-	< 0.01	9.04E-06	2.67E-04
signaling receptor activity (GO:0038023)	749	0	14.15	-	< 0.01	1.22E-06	5.88E-05

Analysis Type:	PANTHER Overrepresentation Test (Released 20200728)
Annotation Version and Release Date:	PANTHER version 15.0 Released 2020-02-14
Analyzed List:	protein.txt (Mycobacterium tuberculosis)
Reference List:	Mycobacterium tuberculosis (all genes in database)
Test Type:	FISHER
Correction:	FDR

PANTHER GO-Slim Biological Process	Mycobacte	protein.txt	protein.txt	protein.txt	protein.txt	protein.txt	protein.txt (FDR)
ribonucleoprotein complex subunit organization (GO:0071826)	11	7	1.46	+	4.81	2.86E-03	4.36E-02
protein-containing complex subunit organization (GO:0043933)	14	8	1.85	+	4.32	2.36E-03	4.12E-02
organelle organization (GO:0006996)	14	8	1.85	+	4.32	2.36E-03	4.01E-02
translational elongation (GO:0006414)	48	19	6.35	+	2.99	1.51E-04	5.13E-03
translation (GO:0006412)	48	19	6.35	+	2.99	1.51E-04	4.86E-03

cellular protein metabolic process (GO:0044267)	67	26	8.86	+	2.93	2.10E-05	9.15E-04
drug metabolic process (GO:0017144)	48	18	6.35	+	2.83	6.41E-04	1.51E-02
peptide biosynthetic process (GO:0043043)	52	19	6.88	+	2.76	4.83E-04	1.18E-02
nucleotide biosynthetic process (GO:0009165)	42	15	5.56	+	2.7	2.52E-03	4.06E-02
peptide metabolic process (GO:0006518)	57	20	7.54	+	2.65	4.39E-04	1.17E-02
nucleoside phosphate biosynthetic process (GO:1901293)	43	15	5.69	+	2.64	2.79E-03	4.36E-02
protein metabolic process (GO:0019538)	80	27	10.58	+	2.55	8.59E-05	3.28E-03
nucleobase-containing small molecule metabolic process (GO:0055086)	67	21	8.86	+	2.37	1.12E-03	2.36E-02

organophosphate biosynthetic process (GO:0090407)	61	19	8.07	+	2.35	2.20E-03	3.96E-02
organonitrogen compound biosynthetic process (GO:1901566)	190	58	25.13	+	2.31	1.15E-07	1.40E-05
carbohydrate derivative biosynthetic process (GO:1901137)	59	18	7.8	+	2.31	3.40E-03	4.95E-02
organophosphate metabolic process (GO:0019637)	86	26	11.38	+	2.29	4.71E-04	1.20E-02
amide biosynthetic process (GO:0043604)	68	20	8.99	+	2.22	3.59E-03	4.99E-02
organic acid biosynthetic process (GO:0016053)	69	20	9.13	+	2.19	3.81E-03	5.17E-02
carboxylic acid biosynthetic process (GO:0046394)	69	20	9.13	+	2.19	3.81E-03	5.06E-02
phosphorus metabolic process (GO:0006793)	95	27	12.57	+	2.15	8.37E-04	1.89E-02

small molecule metabolic process (GO:0044281)	244	69	32.28	+	2.14	7.22E-08	1.10E-05
small molecule biosynthetic process (GO:0044283)	100	28	13.23	+	2.12	1.09E-03	2.37E-02
cellular amino acid metabolic process (GO:0006520)	93	26	12.3	+	2.11	1.25E-03	2.47E-02
carbohydrate derivative metabolic process (GO:1901135)	93	26	12.3	+	2.11	1.25E-03	2.39E-02
phosphate-containing compound metabolic process (GO:0006796)	93	26	12.3	+	2.11	1.25E-03	2.32E-02
cellular amide metabolic process (GO:0043603)	83	23	10.98	+	2.09	3.08E-03	4.59E-02
organic acid metabolic process (GO:0006082)	166	45	21.96	+	2.05	3.85E-05	1.57E-03
cellular catabolic process (GO:0044248)	86	23	11.38	+	2.02	3.81E-03	4.95E-02

organonitrogen compound metabolic process (GO:1901564)	262	69	34.66	+	1.99	7.10E-07	3.62E-05
carboxylic acid metabolic process (GO:0019752)	160	42	21.16	+	1.98	1.77E-04	5.41E-03
oxoacid metabolic process (GO:0043436)	163	42	21.56	+	1.95	2.01E-04	5.86E-03
biosynthetic process (GO:0009058)	361	88	47.75	+	1.84	2.52E-07	2.20E-05
cellular biosynthetic process (GO:0044249)	354	86	46.83	+	1.84	4.40E-07	2.69E-05
organic substance biosynthetic process (GO:1901576)	355	86	46.96	+	1.83	4.62E-07	2.57E-05
cellular nitrogen compound biosynthetic process (GO:0044271)	238	55	31.48	+	1.75	2.25E-04	6.24E-03
cellular process (GO:0009987)	616	141	81.48	+	1.73	6.02E-10	3.68E-07
cellular metabolic process (GO:0044237)	558	126	73.81	+	1.71	2.05E-08	4.18E-06

metabolic process (GO:0008152)	601	133	79.5	+	1.67	1.77E-08	5.41E-06
organic cyclic compound biosynthetic process (GO:1901362)	196	43	25.93	+	1.66	3.51E-03	4.99E-02
organic substance metabolic process (GO:0071704)	558	122	73.81	+	1.65	1.77E-07	1.80E-05
primary metabolic process (GO:0044238)	478	104	63.23	+	1.64	2.23E-06	1.05E-04
nitrogen compound metabolic process (GO:0006807)	436	89	57.67	+	1.54	1.22E-04	4.39E-03
cellular nitrogen compound metabolic process (GO:0034641)	340	69	44.97	+	1.53	1.17E-03	2.39E-02
biological_process (GO:0008150)	745	151	98.54	+	1.53	2.70E-07	1.83E-05
organic cyclic compound metabolic process (GO:1901360)	314	63	41.53	+	1.52	2.46E-03	4.06E-02
Unclassified (UNCLASSIFIED)	3224	374	426.46	-	0.88	2.70E-07	2.06E-05

Analysis Type:	PANTHER Overrepresentation Test (Released 20200728)
Annotation Version and Release Date:	PANTHER version 15.0 Released 2020-02-14
Analyzed List:	protein.txt (Mycobacterium tuberculosis)
Reference List:	Mycobacterium tuberculosis (all genes in database)
Test Type:	FISHER
Correction:	FDR

PANTHER GO-Slim Cellular Component	Mycobacte	protein.txt	protein.txt	protein.txt	protein.txt	protein.txt	protein.txt (FDR)
cytoplasm (GO:0005737)	286	80	37.83	+	2.11	7.17E-09	2.49E-07
intracellular (GO:0005622)	316	86	41.8	+	2.06	4.60E-09	4.79E-07
intracellular part (GO:0044424)	314	85	41.53	+	2.05	6.95E-09	3.62E-07
cytoplasmic part (GO:0044444)	196	49	25.93	+	1.89	1.29E-04	1.67E-03
cytosol (GO:0005829)	161	40	21.3	+	1.88	6.50E-04	7.51E-03
cell part (GO:0044464)	492	105	65.08	+	1.61	4.13E-06	1.07E-04
cell (GO:0005623)	492	105	65.08	+	1.61	4.13E-06	8.60E-05
cellular_component (GO:0005575)	511	105	67.59	+	1.55	1.94E-05	2.89E-04

Unclassified (UNCLASSIFIED)	3458	420	457.41	-	0.92	1.94E-05	3.37E-04

Analysis Type:	PANTHER Overrepresentation Test (Released 20200728)
Annotation Version and Release Date:	PANTHER version 15.0 Released 2020-02-14
Analyzed List:	protein.txt (Mycobacterium tuberculosis)
Reference List:	Mycobacterium tuberculosis (all genes in database)
Test Type:	FISHER
Correction:	FDR

PANTHER GO-Slim Molecular Function	Mycobacte	protein.txt	protein.txt	protein.txt	protein.txt	protein.txt	protein.txt (FDR)
carbon-oxygen lyase activity (GO:0016835)	28	12	3.7	+	3.24	1.45E-03	2.56E-02
structural molecule activity (GO:0005198)	41	17	5.42	+	3.13	2.16E-04	5.91E-03
structural constituent of ribosome (GO:0003735)	41	17	5.42	+	3.13	2.16E-04	5.31E-03
anion binding (GO:0043168)	75	26	9.92	+	2.62	6.35E-05	2.60E-03
small molecule binding (GO:0036094)	84	27	11.11	+	2.43	1.39E-04	4.29E-03
ion binding (GO:0043167)	105	33	13.89	+	2.38	3.81E-05	1.87E-03

oxidoreductase activity (GO:0016491)	144	39	19.05	+	2.05	1.38E-04	4.84E-03
binding (GO:0005488)	276	67	36.51	+	1.84	1.03E-05	6.37E-04
organic cyclic compound binding (GO:0097159)	204	49	26.98	+	1.82	2.52E-04	5.64E-03
heterocyclic compound binding (GO:1901363)	204	49	26.98	+	1.82	2.52E-04	5.17E-03
transferase activity (GO:0016740)	210	47	27.78	+	1.69	1.29E-03	2.45E-02
catalytic activity (GO:0003824)	666	146	88.1	+	1.66	3.84E-09	3.15E-07
molecular_function (GO:0003674)	925	195	122.35	+	1.59	4.12E-11	1.01E-08
Unclassified (UNCLASSIFIED)	3044	330	402.65	-	0.82	4.12E-11	5.07E-09

Analysis Type:	PANTHER Overrepresentation Test (Released 20200728)
Annotation Version and Release Date:	PANTHER version 15.0 Released 2020-02-14
Analyzed List:	protein.txt (Mus musculus)
Reference List:	Mus musculus (all genes in database)
Test Type:	FISHER
Correction:	FDR

PANTHER GO-Slim Biological Process	Mus musc	protein.txt	protein.txt	protein.txt	protein.txt	protein.txt	protein.txt (FDR)
folic acid-containing compound biosynthetic process (GO:0009396)	5	3	0.11	+	26.35	5.65E-04	1.12E-02
heme biosynthetic process (GO:0006783)	7	3	0.16	+	18.82	1.17E-03	2.00E-02
heme metabolic process (GO:0042168)	10	4	0.23	+	17.57	2.04E-04	5.25E-03
porphyrin-containing compound metabolic process (GO:0006778)	10	4	0.23	+	17.57	2.04E-04	5.19E-03

pteridine-containing compound metabolic process (GO:0042558)	13	5	0.3	+	16.89	3.62E-05	1.29E-03
tetrapyrrole metabolic process (GO:0033013)	11	4	0.25	+	15.97	2.73E-04	6.47E-03
cholesterol metabolic process (GO:0008203)	12	4	0.27	+	14.64	3.57E-04	7.93E-03
folic acid-containing compound metabolic process (GO:0006760)	10	3	0.23	+	13.17	2.66E-03	3.70E-02
regulation of ATPase activity (GO:0043462)	11	3	0.25	+	11.98	3.33E-03	4.46E-02
mitochondrial translation (GO:0032543)	15	4	0.34	+	11.71	7.21E-04	1.40E-02
pigment metabolic process (GO:0042440)	16	4	0.36	+	10.98	8.86E-04	1.63E-02
inner mitochondrial membrane organization (GO:0007007)	18	4	0.41	+	9.76	1.29E-03	2.18E-02

coenzyme biosynthetic process (GO:0009108)	41	9	0.93	+	9.64	1.39E-06	7.02E-05
coenzyme metabolic process (GO:0006732)	88	18	2	+	8.98	2.12E-11	3.13E-09
cellular amino acid metabolic process (GO:0006520)	123	25	2.8	+	8.93	2.93E-15	1.01E-12
response to oxidative stress (GO:0006979)	25	5	0.57	+	8.78	4.82E-04	9.86E-03
cofactor biosynthetic process (GO:0051188)	65	13	1.48	+	8.78	1.67E-08	1.28E-06
alpha-amino acid metabolic process (GO:1901605)	77	15	1.75	+	8.55	1.81E-09	1.70E-07
cellular modified amino acid metabolic process (GO:0006575)	67	13	1.53	+	8.52	2.29E-08	1.58E-06
mitochondrial ATP synthesis coupled electron transport (GO:0042775)	21	4	0.48	+	8.36	2.12E-03	3.15E-02

ATP synthesis coupled electron transport (GO:0042773)	22	4	0.5	+		7.98	2.46E-03	3.45E-02
carboxylic acid biosynthetic process (GO:0046394)	84	15	1.91	+		7.84	5.16E-09	4.44E-07
organic acid biosynthetic process (GO:0016053)	84	15	1.91	+		7.84	5.16E-09	4.26E-07
cellular amino acid biosynthetic process (GO:0008652)	34	6	0.77	+		7.75	2.39E-04	5.93E-03
fatty acid biosynthetic process (GO:0006633)	34	6	0.77	+		7.75	2.39E-04	5.86E-03
nucleobase metabolic process (GO:0009112)	23	4	0.52	+		7.64	2.84E-03	3.90E-02
mitochondrial membrane organization (GO:0007006)	23	4	0.52	+		7.64	2.84E-03	3.88E-02
secondary alcohol metabolic process (GO:1902652)	23	4	0.52	+		7.64	2.84E-03	3.85E-02

cofactor metabolic process (GO:0051186)	168	29	3.83	+		7.58	7.68E-16	3.17E-13
glutathione metabolic process (GO:0006749)	35	6	0.8	+		7.53	2.74E-04	6.43E-03
acyl-CoA metabolic process (GO:0006637)	35	6	0.8	+		7.53	2.74E-04	6.36E-03
respiratory electron transport chain (GO:0022904)	30	5	0.68	+		7.32	1.00E-03	1.82E-02
electron transport chain (GO:0022900)	30	5	0.68	+		7.32	1.00E-03	1.80E-02
small molecule biosynthetic process (GO:0044283)	155	25	3.53	+		7.08	3.02E-13	6.93E-11
mitochondrial gene expression (GO:0140053)	25	4	0.57	+		7.03	3.71E-03	4.81E-02
monocarboxylic acid biosynthetic process (GO:0072330)	38	6	0.87	+		6.93	4.07E-04	8.94E-03
sulfur compound metabolic process (GO:0006790)	118	18	2.69	+		6.7	1.45E-09	1.42E-07

carboxylic acid catabolic process (GO:0046395)	73	11	1.66	+	6.62	2.57E-06	1.20E-04
organic acid catabolic process (GO:0016054)	73	11	1.66	+	6.62	2.57E-06	1.18E-04
small molecule catabolic process (GO:0044282)	108	15	2.46	+	6.1	1.03E-07	6.65E-06
hexose metabolic process (GO:0019318)	36	5	0.82	+	6.1	2.08E-03	3.10E-02
carboxylic acid metabolic process (GO:0019752)	331	44	7.54	+	5.84	1.83E-19	1.89E-16
oxoacid metabolic process (GO:0043436)	342	44	7.79	+	5.65	5.65E-19	3.89E-16
organic acid metabolic process (GO:0006082)	355	44	8.08	+	5.44	2.03E-18	1.05E-15
small molecule metabolic process (GO:0044281)	587	71	13.37	+	5.31	1.05E-28	2.18E-25
ribosomal large subunit biogenesis (GO:0042273)	63	7	1.43	+	4.88	9.30E-04	1.70E-02

cellular amide metabolic process (GO:0043603)	369	39	8.4	+	4.64	2.21E-14	6.51E-12
purine-containing compound metabolic process (GO:0072521)	152	16	3.46	+	4.62	1.19E-06	6.28E-05
peptide biosynthetic process (GO:0043043)	219	23	4.99	+	4.61	5.84E-09	4.64E-07
monocarboxylic acid metabolic process (GO:0032787)	181	19	4.12	+	4.61	1.24E-07	7.55E-06
fatty acid metabolic process (GO:0006631)	125	13	2.85	+	4.57	1.33E-05	5.38E-04
nucleobase-containing small molecule metabolic process (GO:0055086)	202	21	4.6	+	4.57	3.16E-08	2.10E-06
cellular respiration (GO:0045333)	58	6	1.32	+	4.54	2.98E-03	4.02E-02
amide biosynthetic process (GO:0043604)	252	26	5.74	+	4.53	8.36E-10	9.58E-08

translational elongation (GO:0006414)	216	22	4.92	+	4.47	2.08E-08	1.53E-06
translation (GO:0006412)	216	22	4.92	+	4.47	2.08E-08	1.48E-06
peptide metabolic process (GO:0006518)	286	29	6.51	+	4.45	1.27E-10	1.55E-08
alcohol metabolic process (GO:0006066)	70	7	1.59	+	4.39	1.63E-03	2.59E-02
nucleotide metabolic process (GO:0009117)	163	16	3.71	+	4.31	2.74E-06	1.23E-04
organic hydroxy compound metabolic process (GO:1901615)	94	9	2.14	+	4.2	4.93E-04	9.98E-03
nucleoside phosphate metabolic process (GO:0006753)	168	16	3.83	+	4.18	3.92E-06	1.72E-04
purine ribonucleotide metabolic process (GO:0009150)	130	12	2.96	+	4.05	8.27E-05	2.71E-03
tRNA metabolic process (GO:0006399)	99	9	2.25	+	3.99	6.97E-04	1.37E-02

ribonucleotide metabolic process (GO:0009259)	135	12	3.07	+	3.9	1.15E-04	3.40E-03
purine nucleotide metabolic process (GO:0006163)	138	12	3.14	+	3.82	1.40E-04	4.00E-03
organic cyclic compound catabolic process (GO:1901361)	151	13	3.44	+	3.78	8.18E-05	2.72E-03
drug metabolic process (GO:0017144)	189	16	4.3	+	3.72	1.55E-05	6.14E-04
ribose phosphate metabolic process (GO:0019693)	143	12	3.26	+	3.69	1.90E-04	5.03E-03
organonitrogen compound biosynthetic process (GO:1901566)	589	49	13.41	+	3.65	4.90E-14	1.26E-11
aromatic compound catabolic process (GO:0019439)	139	10	3.17	+	3.16	1.90E-03	2.91E-02
heterocycle catabolic process (GO:0046700)	140	10	3.19	+	3.14	2.00E-03	3.03E-02

oxidation-reduction process (GO:0055114)	220	15	5.01	+	2.99	2.71E-04	6.49E-03
ribosome biogenesis (GO:0042254)	176	12	4.01	+	2.99	1.08E-03	1.86E-02
ncRNA metabolic process (GO:0034660)	240	14	5.47	+	2.56	1.77E-03	2.77E-02
organophosphate metabolic process (GO:0019637)	312	17	7.1	+	2.39	1.79E-03	2.76E-02
organic substance catabolic process (GO:1901575)	703	36	16.01	+	2.25	1.16E-05	4.88E-04
lipid metabolic process (GO:0006629)	401	20	9.13	+	2.19	2.07E-03	3.12E-02
catabolic process (GO:0009056)	888	42	20.22	+	2.08	1.86E-05	7.11E-04
cellular catabolic process (GO:0044248)	765	33	17.42	+	1.89	8.17E-04	1.52E-02
organonitrogen compound metabolic process (GO:1901564)	2527	106	57.54	+	1.84	9.87E-10	1.07E-07
biosynthetic process (GO:0009058)	2006	76	45.68	+	1.66	1.61E-05	6.26E-04

cellular biosynthetic process (GO:0044249)	1964	73	44.72	+	1.63	5.25E-05	1.84E-03
organic substance biosynthetic process (GO:1901576)	1986	73	45.22	+	1.61	7.99E-05	2.70E-03
cellular nitrogen compound metabolic process (GO:0034641)	2415	83	54.99	+	1.51	2.29E-04	5.76E-03
cellular metabolic process (GO:0044237)	4357	144	99.21	+	1.45	2.54E-06	1.22E-04
metabolic process (GO:0008152)	4857	158	110.6	+	1.43	1.29E-06	6.67E-05
organic substance metabolic process (GO:0071704)	4680	149	106.57	+	1.4	1.31E-05	5.40E-04
nitrogen compound metabolic process (GO:0006807)	4212	127	95.91	+	1.32	7.27E-04	1.40E-02
primary metabolic process (GO:0044238)	4458	131	101.51	+	1.29	1.69E-03	2.66E-02
regulation of metabolic process (GO:0019222)	1956	24	44.54	-	0.54	7.71E-04	1.45E-02

biological regulation (GO:0065007)	4862	57	110.71	-	0.51	1.04E-09	1.07E-07
regulation of cellular metabolic process (GO:0031323)	1821	21	41.47	-	0.51	5.03E-04	1.01E-02
regulation of primary metabolic process (GO:0080090)	1804	20	41.08	-	0.49	2.57E-04	6.25E-03
regulation of gene expression (GO:0010468)	1368	15	31.15	-	0.48	1.79E-03	2.78E-02
regulation of nitrogen compound metabolic process (GO:0051171)	1782	19	40.58	-	0.47	1.66E-04	4.45E-03
regulation of macromolecule metabolic process (GO:0060255)	1878	20	42.76	-	0.47	1.20E-04	3.48E-03
regulation of cellular biosynthetic process (GO:0031326)	1234	12	28.1	-	0.43	1.01E-03	1.80E-02
response to stimulus (GO:0050896)	3351	32	76.31	-	0.42	2.11E-09	1.89E-07

multicellular organismal process (GO:0032501)	1378	13	31.38	-	0.41	3.23E-04	7.25E-03
RNA biosynthetic process (GO:0032774)	1166	11	26.55	-	0.41	1.06E-03	1.83E-02
regulation of biological process (GO:0050789)	4413	41	100.49	-	0.41	5.54E-13	1.14E-10
regulation of cellular process (GO:0050794)	4160	38	94.73	-	0.4	2.03E-12	3.81E-10
regulation of cellular macromolecule biosynthetic process (GO:2000112)	1216	11	27.69	-	0.4	4.50E-04	9.58E-03
regulation of macromolecule biosynthetic process (GO:0010556)	1223	11	27.85	-	0.39	4.57E-04	9.63E-03
multicellular organism development (GO:0007275)	828	7	18.85	-	0.37	3.73E-03	4.82E-02
anatomical structure development (GO:0048856)	1076	9	24.5	-	0.37	4.65E-04	9.69E-03

developmental process (GO:0032502)	1209	10	27.53	-	0.36	1.93E-04	5.05E-03
regulation of RNA metabolic process (GO:0051252)	1222	10	27.83	-	0.36	1.43E-04	3.93E-03
regulation of nucleobase-containing compound metabolic process (GO:0019219)	1263	10	28.76	-	0.35	7.88E-05	2.71E-03
nucleic acid-templated transcription (GO:0097659)	1159	9	26.39	-	0.34	1.42E-04	4.02E-03
transcription, DNA-templated (GO:0006351)	1159	9	26.39	-	0.34	1.42E-04	3.96E-03
cellular response to stimulus (GO:0051716)	2758	20	62.8	-	0.32	1.00E-10	1.29E-08
regulation of transcription, DNA-templated (GO:0006355)	1117	8	25.44	-	0.31	1.03E-04	3.19E-03

regulation of nucleic acid-templated transcription (GO:1903506)	1117	8	25.44	-	0.31	1.03E-04	3.14E-03
regulation of RNA biosynthetic process (GO:2001141)	1117	8	25.44	-	0.31	1.03E-04	3.09E-03
regulation of transcription by RNA polymerase II (GO:0006357)	745	5	16.96	-	0.29	1.44E-03	2.41E-02
positive regulation of cellular process (GO:0048522)	1346	9	30.65	-	0.29	6.29E-06	2.70E-04
transcription by RNA polymerase II (GO:0006366)	772	5	17.58	-	0.28	7.47E-04	1.43E-02
positive regulation of biological process (GO:0048518)	1559	9	35.5	-	0.25	1.72E-07	9.88E-06
signal transduction (GO:0007165)	2229	12	50.76	-	0.24	4.13E-11	5.68E-09
signaling (GO:0023052)	2365	12	53.85	-	0.22	3.31E-12	5.69E-10

cell communication (GO:0007154)	2367	12	53.9	-	0.22	3.35E-12	5.32E-10
G protein-coupled receptor signaling pathway (GO:0007186)	682	3	15.53	-	0.19	2.99E-04	6.86E-03
positive regulation of nucleobase-containing compound metabolic process (GO:0045935)	461	2	10.5	-	0.19	3.63E-03	4.74E-02
multi-organism process (GO:0051704)	570	2	12.98	-	0.15	4.49E-04	9.65E-03
regulation of signal transduction (GO:0009966)	627	2	14.28	-	0.14	1.57E-04	4.27E-03
response to external stimulus (GO:0009605)	686	2	15.62	-	0.13	3.58E-05	1.34E-03
regulation of response to stimulus (GO:0048583)	1032	3	23.5	-	0.13	2.26E-07	1.26E-05
regulation of cell communication (GO:0010646)	691	2	15.73	-	0.13	3.59E-05	1.32E-03

regulation of signaling (GO:0023051)	693	2	15.78	-	0.13	3.62E-05	1.31E-03
regulation of immune system process (GO:0002682)	389	1	8.86	-	0.11	2.72E-03	3.77E-02
cell surface receptor signaling pathway (GO:0007166)	925	2	21.06	-	0.09	2.94E-07	1.60E-05
defense response (GO:0006952)	477	1	10.86	-	0.09	4.32E-04	9.38E-03
immune system process (GO:0002376)	839	1	19.11	-	0.05	1.46E-07	8.64E-06
membrane invagination (GO:0010324)	303	0	6.9	-	< 0.01	2.38E-03	3.47E-02
positive regulation of cell communication (GO:0010647)	292	0	6.65	-	< 0.01	2.25E-03	3.32E-02
response to external biotic stimulus (GO:0043207)	429	0	9.77	-	< 0.01	9.68E-05	3.12E-03
positive regulation of signaling (GO:0023056)	293	0	6.67	-	< 0.01	2.26E-03	3.30E-02

defense response to bacterium (GO:0042742)	273	0	6.22	-	< 0.01	3.33E-03	4.44E-02
response to other organism (GO:0051707)	429	0	9.77	-	< 0.01	9.68E-05	3.07E-03
regulation of intracellular signal transduction (GO:1902531)	368	0	8.38	-	< 0.01	4.81E-04	9.93E-03
innate immune response (GO:0045087)	319	0	7.26	-	< 0.01	1.59E-03	2.59E-02
positive regulation of immune system process (GO:0002684)	358	0	8.15	-	< 0.01	7.63E-04	1.44E-02
negative regulation of nucleobase-containing compound metabolic process (GO:0045934)	329	0	7.49	-	< 0.01	1.03E-03	1.82E-02
response to bacterium (GO:0009617)	317	0	7.22	-	< 0.01	1.57E-03	2.57E-02
nervous system process (GO:0050877)	320	0	7.29	-	< 0.01	1.61E-03	2.59E-02

response to biotic stimulus (GO:0009607)	430	0	9.79	-	< 0.01	9.70E-05	3.03E-03
negative regulation of nucleic acid-templated transcription (GO:1903507)	305	0	6.95	-	< 0.01	2.43E-03	3.49E-02
positive regulation of response to stimulus (GO:0048584)	612	0	13.94	-	< 0.01	1.99E-06	9.80E-05
activation of immune response (GO:0002253)	278	0	6.33	-	< 0.01	3.36E-03	4.45E-02
immune effector process (GO:0002252)	309	0	7.04	-	< 0.01	1.52E-03	2.53E-02
humoral immune response (GO:0006959)	279	0	6.35	-	< 0.01	3.37E-03	4.44E-02
immune response (GO:0006955)	729	0	16.6	-	< 0.01	1.19E-07	7.46E-06
negative regulation of RNA metabolic process (GO:0051253)	316	0	7.2	-	< 0.01	1.56E-03	2.57E-02

negative regulation of transcription, DNA-templated (GO:0045892)	305	0	6.95	-	< 0.01	2.43E-03	3.46E-02
positive regulation of immune response (GO:0050778)	321	0	7.31	-	< 0.01	1.62E-03	2.60E-02
regulation of immune response (GO:0050776)	331	0	7.54	-	< 0.01	1.04E-03	1.82E-02
negative regulation of RNA biosynthetic process (GO:1902679)	305	0	6.95	-	< 0.01	2.43E-03	3.44E-02
endocytosis (GO:0006897)	303	0	6.9	-	< 0.01	2.38E-03	3.44E-02
defense response to other organism (GO:0098542)	381	0	8.68	-	< 0.01	3.18E-04	7.21E-03

Analysis Type:	PANTHER Overrepresentation Test (Released 20200728)						
Annotation Version and Release Date:	PANTHER version 15.0 Released 2020-02-14						
Analyzed List:	protein.txt (Mus musculus)						
Reference List:	Mus musculus (all genes in database)						
Test Type:	FISHER						
Correction:	FDR						
PANTHER GO-Slim Cellular Component	Mus musc	protein.txt	protein.txt	protein.txt	protein.txt	protein.txt	protein.txt (FDR)
mitochondrial respiratory chain complex I (GO:0005747)	24	11	0.55	+	20.13	1.54E-10	3.48E-09
respiratory chain complex I (GO:0045271)	27	12	0.61	+	19.52	2.95E-11	9.03E-10
NADH dehydrogenase complex (GO:0030964)	28	12	0.64	+	18.82	4.13E-11	1.19E-09
oligosaccharyltransferase complex (GO:0008250)	8	3	0.18	+	16.47	1.58E-03	1.55E-02
respiratory chain complex (GO:0098803)	44	14	1	+	13.97	2.55E-11	8.28E-10

mitochondrial respirasome (GO:0005746)	41	13	0.93	+	13.92	1.37E-10	3.23E-09
oxidoreductase complex (GO:1990204)	43	13	0.98	+	13.28	2.24E-10	4.85E-09
respirasome (GO:0070469)	48	14	1.09	+	12.81	6.73E-11	1.75E-09
inner mitochondrial membrane protein complex (GO:0098800)	69	17	1.57	+	10.82	6.01E-12	3.13E-10
ER to Golgi transport vesicle membrane (GO:0012507)	14	3	0.32	+	9.41	5.91E-03	4.46E-02
mitochondrial inner membrane (GO:0005743)	118	24	2.69	+	8.93	1.05E-14	1.09E-12
mitochondrial protein complex (GO:0098798)	115	23	2.62	+	8.78	5.15E-14	3.83E-12
large ribosomal subunit (GO:0015934)	86	16	1.96	+	8.17	9.40E-10	1.88E-08
organelle inner membrane (GO:0019866)	132	24	3.01	+	7.98	9.09E-14	5.25E-12

mitochondrial membrane part (GO:0044455)	106	19	2.41	+	7.87	4.39E-11	1.20E-09
cytosolic large ribosomal subunit (GO:0022625)	64	11	1.46	+	7.55	8.10E-07	1.36E-05
mitochondrial membrane (GO:0031966)	174	27	3.96	+	6.81	7.69E-14	5.00E-12
cytosolic ribosome (GO:0022626)	104	16	2.37	+	6.76	1.06E-08	1.98E-07
mitochondrial envelope (GO:0005740)	189	29	4.3	+	6.74	1.17E-14	1.01E-12
ribosome (GO:0005840)	151	23	3.44	+	6.69	7.73E-12	3.65E-10
mitochondrial part (GO:0044429)	239	36	5.44	+	6.61	1.14E-17	1.48E-15
ribosomal subunit (GO:0044391)	143	21	3.26	+	6.45	1.16E-10	2.88E-09
mitochondrial matrix (GO:0005759)	56	8	1.28	+	6.27	8.47E-05	1.10E-03
mitochondrion (GO:0005739)	442	61	10.06	+	6.06	1.87E-27	9.70E-25
organellar ribosome (GO:0000313)	30	4	0.68	+	5.86	6.64E-03	4.86E-02
mitochondrial ribosome (GO:0005761)	30	4	0.68	+	5.86	6.64E-03	4.79E-02

focal adhesion (GO:0005925)	38	5	0.87	+	5.78	2.57E-03	2.23E-02
cytosolic small ribosomal subunit (GO:0022627)	40	5	0.91	+	5.49	3.15E-03	2.52E-02
cytosolic part (GO:0044445)	162	19	3.69	+	5.15	2.51E-08	4.49E-07
envelope (GO:0031975)	283	30	6.44	+	4.66	2.23E-11	8.29E-10
organelle envelope (GO:0031967)	283	30	6.44	+	4.66	2.23E-11	7.74E-10
organelle membrane (GO:0031090)	665	45	15.14	+	2.97	3.43E-10	7.13E-09
cytosol (GO:0005829)	753	46	17.15	+	2.68	4.59E-09	8.84E-08
ribonucleoprotein complex (GO:1990904)	463	28	10.54	+	2.66	6.23E-06	9.53E-05
endoplasmic reticulum membrane (GO:0005789)	217	13	4.94	+	2.63	2.04E-03	1.87E-02
nuclear outer membrane-endoplasmic reticulum membrane network (GO:0042175)	219	13	4.99	+	2.61	2.21E-03	1.98E-02

endoplasmic reticulum part (GO:0044432)	251	14	5.72	+		2.45	2.61E-03	2.23E-02
membrane protein complex (GO:0098796)	541	30	12.32	+		2.44	1.66E-05	2.34E-04
cytoplasmic part (GO:0044444)	2902	148	66.08	+		2.24	4.15E-21	7.19E-19
endoplasmic reticulum (GO:0005783)	450	21	10.25	+		2.05	2.45E-03	2.16E-02
catalytic complex (GO:1902494)	772	35	17.58	+		1.99	2.09E-04	2.53E-03
cytoplasm (GO:0005737)	4183	189	95.25	+		1.98	6.93E-22	1.80E-19
intracellular organelle part (GO:0044446)	2644	98	60.21	+		1.63	1.68E-06	2.72E-05
organelle part (GO:0044422)	2726	99	62.07	+		1.59	4.27E-06	6.73E-05
intracellular part (GO:0044424)	6691	225	152.36	+		1.48	2.15E-11	8.62E-10
intracellular (GO:0005622)	6721	226	153.05	+		1.48	1.66E-11	7.18E-10
protein-containing complex (GO:0032991)	2747	90	62.55	+		1.44	4.99E-04	5.77E-03
intracellular organelle (GO:0043229)	5332	166	121.42	+		1.37	9.70E-06	1.40E-04

organelle (GO:0043226)	5434	169	123.74	+	1.37	8.61E-06	1.28E-04
membrane-bounded organelle (GO:0043227)	4721	139	107.5	+	1.29	9.90E-04	1.10E-02
intracellular membrane-bounded organelle (GO:0043231)	4662	137	106.16	+	1.29	1.37E-03	1.39E-02
cell part (GO:0044464)	8474	237	192.96	+	1.23	8.34E-05	1.14E-03
cell (GO:0005623)	8474	237	192.96	+	1.23	8.34E-05	1.11E-03
cellular_component (GO:0005575)	9552	251	217.51	+	1.15	3.18E-03	2.47E-02
Unclassified (UNCLASSIFIED)	12713	256	289.49	-	0.88	3.18E-03	2.50E-02
cell periphery (GO:0071944)	3312	46	75.42	-	0.61	1.38E-04	1.75E-03
plasma membrane (GO:0005886)	3243	45	73.85	-	0.61	1.98E-04	2.45E-03
nucleus (GO:0005634)	2974	41	67.72	-	0.61	3.27E-04	3.87E-03
integral component of membrane (GO:0016021)	1114	12	25.37	-	0.47	4.86E-03	3.72E-02
intrinsic component of membrane (GO:0031224)	1141	12	25.98	-	0.46	2.87E-03	2.33E-02

extracellular region (GO:0005576)	1253	13	28.53	-	0.46	1.60E-03	1.54E-02
extracellular region part (GO:0044421)	1253	13	28.53	-	0.46	1.60E-03	1.51E-02
extracellular space (GO:0005615)	1169	11	26.62	-	0.41	1.07E-03	1.16E-02
integral component of plasma membrane (GO:0005887)	770	6	17.53	-	0.34	2.70E-03	2.27E-02
intrinsic component of plasma membrane (GO:0031226)	777	6	17.69	-	0.34	2.76E-03	2.28E-02
plasma membrane region (GO:0098590)	582	4	13.25	-	0.3	6.38E-03	4.74E-02
plasma membrane part (GO:0044459)	1622	11	36.93	-	0.3	7.74E-07	1.34E-05
plasma membrane bounded cell projection (GO:0120025)	497	2	11.32	-	0.18	1.82E-03	1.69E-02
cell projection (GO:0042995)	513	2	11.68	-	0.17	1.28E-03	1.35E-02
cell surface (GO:0009986)	421	1	9.59	-	0.1	1.30E-03	1.35E-02

neuron part (GO:0097458)	438	1	9.97	-	0.1	8.97E-04	1.01E-02
external side of plasma membrane (GO:0009897)	318	0	7.24	-	< 0.01	1.58E-03	1.58E-02

Analysis Type:	PANTHER Overrepresentation Test (Released 20200728)
Annotation Version and Release Date:	PANTHER version 15.0 Released 2020-02-14
Analyzed List:	protein.txt (Mus musculus)
Reference List:	Mus musculus (all genes in database)
Test Type:	FISHER
Correction:	FDR

PANTHER GO-Slim Molecular Function	Mus musc	protein.txt	protein.txt	protein.txt	protein.txt	protein.txt	protein.txt
oligosaccharyl transferase activity (GO:0004576)	5	3	0.11	+	26.35	5.65E-04	1.43E-02
flavin adenine dinucleotide binding (GO:0050660)	30	8	0.68	+	11.71	1.55E-06	6.87E-05
cholesterol binding (GO:0015485)	19	4	0.43	+	9.25	1.54E-03	2.91E-02
sterol binding (GO:0032934)	26	5	0.59	+	8.45	5.65E-04	1.50E-02
structural constituent of ribosome (GO:0003735)	128	21	2.91	+	7.2	1.83E-11	2.43E-09

oxidoreductase activity, acting on the CH-OH group of donors, NAD or NADP as acceptor (GO:0016616)	76	11	1.73	+	6.36	3.64E-06	1.38E-04
steroid binding (GO:0005496)	43	6	0.98	+	6.13	7.34E-04	1.62E-02
ligase activity (GO:0016874)	88	12	2	+	5.99	2.32E-06	9.47E-05
cofactor binding (GO:0048037)	169	22	3.85	+	5.72	3.35E-10	2.54E-08
structural molecule activity (GO:0005198)	218	23	4.96	+	4.63	5.40E-09	3.58E-07
oxidoreductase activity (GO:0016491)	443	45	10.09	+	4.46	7.71E-16	4.09E-13
catalytic activity, acting on a tRNA (GO:0140101)	79	8	1.8	+	4.45	7.16E-04	1.65E-02
actin filament binding (GO:0051015)	84	8	1.91	+	4.18	1.04E-03	2.12E-02
nucleoside phosphate binding (GO:1901265)	220	20	5.01	+	3.99	4.90E-07	2.60E-05
nucleotide binding (GO:0000166)	220	20	5.01	+	3.99	4.90E-07	2.37E-05

small molecule binding (GO:0036094)	358	29	8.15	+	3.56	1.46E-08	8.60E-07
actin binding (GO:0003779)	129	10	2.94	+	3.4	1.13E-03	2.22E-02
anion binding (GO:0043168)	473	26	10.77	+	2.41	7.29E-05	2.42E-03
ion binding (GO:0043167)	711	34	16.19	+	2.1	1.11E-04	3.47E-03
catalytic activity (GO:0003824)	3715	156	84.59	+	1.84	1.25E-14	3.33E-12
organic cyclic compound binding (GO:0097159)	1680	60	38.26	+	1.57	6.83E-04	1.65E-02
molecular_function (GO:0003674)	9001	247	204.96	+	1.21	2.06E-04	5.77E-03
Unclassified (UNCLASSIFIED)	13264	260	302.04	-	0.86	2.06E-04	6.09E-03
transcription regulator activity (GO:0140110)	772	6	17.58	-	0.34	2.72E-03	4.81E-02
DNA-binding transcription factor activity (GO:0003700)	618	3	14.07	-	0.21	8.01E-04	1.70E-02
signaling receptor binding (GO:0005102)	734	2	16.71	-	0.12	1.79E-05	6.35E-04

G protein-coupled receptor activity (GO:0004930)	405	1	9.22	-	0.11	1.88E-03	3.44E-02
molecular transducer activity (GO:0060089)	1349	3	30.72	-	0.1	2.46E-10	2.18E-08
transmembrane signaling receptor activity (GO:0004888)	1182	1	26.92	-	0.04	8.50E-11	9.03E-09
signaling receptor activity (GO:0038023)	1282	1	29.19	-	0.03	7.30E-12	1.29E-09

(FDR)

Analysis Type:	PANTHER Overrepresentation Test (Released 20200728)
Annotation Version and Release Date:	PANTHER version 15.0 Released 2020-02-14
Analyzed List:	protein.txt (Saccharomyces cerevisiae)
Reference List:	Saccharomyces cerevisiae (all genes in database)
Test Type:	FISHER
Correction:	FDR

PANTHER GO-Slim Biological Process	Saccharom	protein.txt	protein.txt	protein.txt	protein.txt	protein.txt	protein.txt (FDR)
small molecule metabolic process (GO:0044281)	354	49	16.62	+	2.95	5.93E-11	8.06E-08
organonitrogen compound biosynthetic process (GO:1901566)	446	53	20.95	+	2.53	1.47E-09	9.97E-07
oxoacid metabolic process (GO:0043436)	194	31	9.11	+	3.4	1.52E-08	4.14E-06
carboxylic acid metabolic process (GO:0019752)	190	31	8.92	+	3.47	9.85E-09	4.46E-06
organic acid metabolic process (GO:0006082)	194	31	9.11	+	3.4	1.52E-08	5.17E-06

cellular amino acid metabolic process (GO:0006520)	129	24	6.06	+	3.96	5.85E-08	1.33E-05
organonitrogen compound metabolic process (GO:1901564)	892	75	41.89	+	1.79	7.15E-07	1.39E-04
cellular amino acid biosynthetic process (GO:0008652)	60	15	2.82	+	5.32	8.28E-07	1.41E-04
peptide metabolic process (GO:0006518)	174	25	8.17	+	3.06	2.37E-06	3.58E-04
peptide biosynthetic process (GO:0043043)	162	23	7.61	+	3.02	7.29E-06	6.60E-04
organic acid biosynthetic process (GO:0016053)	83	16	3.9	+	4.1	6.84E-06	6.64E-04
translation (GO:0006412)	160	23	7.51	+	3.06	6.06E-06	6.86E-04
carboxylic acid biosynthetic process (GO:0046394)	83	16	3.9	+	4.1	6.84E-06	7.15E-04
cellular amide metabolic process (GO:0043603)	214	27	10.05	+	2.69	8.44E-06	7.17E-04

amide biosynthetic process (GO:0043604)	183	25	8.59	+	2.91	5.32E-06	7.23E-04
translational elongation (GO:0006414)	160	23	7.51	+	3.06	6.06E-06	7.49E-04
small molecule biosynthetic process (GO:0044283)	139	20	6.53	+	3.06	2.46E-05	1.97E-03
organic substance biosynthetic process (GO:1901576)	796	62	37.38	+	1.66	8.48E-05	6.41E-03
biosynthetic process (GO:0009058)	810	62	38.04	+	1.63	1.38E-04	9.87E-03
cellular biosynthetic process (GO:0044249)	797	61	37.43	+	1.63	1.74E-04	1.18E-02
cellular process (GO:0009987)	2163	132	101.58	+	1.3	3.03E-04	1.96E-02
protein folding (GO:0006457)	53	10	2.49	+	4.02	4.35E-04	2.69E-02

Analysis Type:	PANTHER Overrepresentation Test (Released 20200728)
Annotation Version and Release Date:	PANTHER version 15.0 Released 2020-02-14
Analyzed List:	protein.txt (Saccharomyces cerevisiae)
Reference List:	Saccharomyces cerevisiae (all genes in database)
Test Type:	FISHER
Correction:	FDR

PANTHER GO-Slim Cellular Component	Saccharom	protein.txt	protein.txt	protein.txt	protein.txt	protein.txt	protein.txt (FDR)
proteasome regulatory particle, lid subcomplex (GO:0008541)	7	4	0.33	+	12.17	1.02E-03	2.45E-02
proteasome accessory complex (GO:0022624)	14	5	0.66	+	7.6	1.21E-03	2.61E-02
proteasome regulatory particle (GO:0005838)	14	5	0.66	+	7.6	1.21E-03	2.48E-02
proton-transporting two-sector ATPase complex (GO:0016469)	16	5	0.75	+	6.65	1.97E-03	3.51E-02

endopeptidase complex (GO:1905369)	29	9	1.36	+	6.61	3.35E-05	1.24E-03
proteasome complex (GO:0000502)	29	9	1.36	+	6.61	3.35E-05	1.14E-03
peptidase complex (GO:1905368)	43	9	2.02	+	4.46	4.35E-04	1.19E-02
cytosolic small ribosomal subunit (GO:0022627)	51	10	2.4	+	4.18	3.32E-04	9.71E-03
small ribosomal subunit (GO:0015935)	68	11	3.19	+	3.44	7.23E-04	1.85E-02
cytosolic part (GO:0044445)	145	23	6.81	+	3.38	1.37E-06	9.31E-05
cytosolic ribosome (GO:0022626)	123	17	5.78	+	2.94	1.57E-04	4.93E-03
cytosol (GO:0005829)	406	53	19.07	+	2.78	6.26E-11	1.28E-08
ribosomal subunit (GO:0044391)	157	18	7.37	+	2.44	1.09E-03	2.47E-02
ribosome (GO:0005840)	164	18	7.7	+	2.34	1.47E-03	2.86E-02
cytoplasmic part (GO:0044444)	1197	104	56.21	+	1.85	1.43E-10	1.94E-08
cytoplasm (GO:0005737)	1627	134	76.41	+	1.75	1.26E-12	5.16E-10
protein-containing complex (GO:0032991)	1139	76	53.49	+	1.42	1.50E-03	2.78E-02

intracellular part (GO:0044424)	2319	154	108.91	+	1.41	1.36E-07	1.39E-05
intracellular (GO:0005622)	2329	154	109.38	+	1.41	1.95E-07	1.59E-05
cell part (GO:0044464)	2515	156	118.11	+	1.32	1.13E-05	6.59E-04
cell (GO:0005623)	2515	156	118.11	+	1.32	1.13E-05	5.76E-04
cellular_component (GO:0005575)	2596	159	121.92	+	1.3	1.64E-05	7.48E-04
Unclassified (UNCLASSIFIED)	3430	124	161.08	-	0.77	1.64E-05	6.73E-04

Analysis Type:	PANTHER Overrepresentation Test (Released 20200728)
Annotation Version and Release Date:	PANTHER version 15.0 Released 2020-02-14
Analyzed List:	protein.txt (Saccharomyces cerevisiae)
Reference List:	Saccharomyces cerevisiae (all genes in database)
Test Type:	FISHER
Correction:	FDR

PANTHER GO-Slim Molecular Function	Saccharom	protein.txt	protein.txt	protein.txt	protein.txt	protein.txt	protein.txt
chaperone binding (GO:0051087)	8	5	0.38	+	13.31	1.68E-04	1.45E-02
translation initiation factor activity (GO:0003743)	16	6	0.75	+	7.98	3.14E-04	2.27E-02
translation regulator activity (GO:0045182)	30	7	1.41	+	4.97	1.09E-03	3.96E-02
endopeptidase activity (GO:0004175)	42	9	1.97	+	4.56	3.74E-04	2.32E-02
ATPase-coupled transmembrane transporter activity (GO:0042626)	40	8	1.88	+	4.26	1.17E-03	3.91E-02

isomerase activity (GO:0016853)	52	10	2.44	+	4.09	3.81E-04	2.07E-02
primary active transmembrane transporter activity (GO:0015399)	42	8	1.97	+	4.06	1.54E-03	4.77E-02
ligase activity (GO:0016874)	68	11	3.19	+	3.44	7.23E-04	3.14E-02
oxidoreductase activity (GO:0016491)	185	20	8.69	+	2.3	8.83E-04	3.48E-02
RNA binding (GO:0003723)	285	28	13.38	+	2.09	3.82E-04	1.84E-02
binding (GO:0005488)	981	72	46.07	+	1.56	1.18E-04	1.28E-02
catalytic activity (GO:0003824)	1460	105	68.57	+	1.53	2.83E-06	4.10E-04
molecular_function (GO:0003674)	2468	168	115.91	+	1.45	1.53E-09	3.32E-07
Unclassified (UNCLASSIFIED)	3558	115	167.09	-	0.69	1.53E-09	6.65E-07

(FDR)
